# Integrating Chinese medicine into mainstream cancer therapies: a promising future

**DOI:** 10.3389/fonc.2024.1412370

**Published:** 2024-06-18

**Authors:** Baoyi Ni, Kaiyuan Xue, Jia Wang, Jilai Zhou, Lankang Wang, Xinmiao Wang, Ting Liu, Naijing Ye, Jiakang Jiang

**Affiliations:** ^1^ Heilongjiang University of Chinese Medicine, Harbin, China; ^2^ The First Affiliated Hospital of Heilongjiang University of Chinese Medicine, Harbin, China; ^3^ Hongqi Hospital of Mudanjiang Medical University, Mudanjiang, China; ^4^ Guang’anmen Hospital, China Academy of Chinese Medical Sciences, Beijing, China; ^5^ Hospital of Chengdu University of Traditional Chinese Medicine, Chengdu, China

**Keywords:** Chinese herb monomer, Chinese patent medicine, malignant tumors, adjuvant therapy, adverse effects, drug resistance

## Abstract

Malignant tumors are complex systemic chronic diseases and one of the major causes of human mortality. Targeted therapy, chemotherapy, immunotherapy, and radiotherapy are examples of mainstream allopathic medicine treatments that effective for intermediate and advanced malignant tumors. The ongoing use of conventional allopathic medicine has resulted in adverse responses and drug resistance, which have hampered its efficacy. As an important component of complementary and alternative medicine, Chinese medicine has been found to have antitumor effects and has played an important role in enhancing the therapeutic sensitivity of mainstream allopathic medicine, reducing the incidence of adverse events and improving immune-related functions. The combined application of adjuvant Chinese medicine and mainstream allopathic medicine has begun to gain acceptance and is gradually used in the field of antitumor therapy. Traditional natural medicines and their active ingredients, as well as Chinese patent medicines, have been proven to have excellent therapeutic efficacy and good safety in the treatment of various malignant tumors. This paper focuses on the mechanism of action and research progress of combining the above drugs with mainstream allopathic medicine to increase therapeutic sensitivity, alleviate drug resistance, reduce adverse reactions, and improve the body’s immune function. To encourage the clinical development and use of Chinese herb adjuvant therapy as well as to provide ideas and information for creating safer and more effective anticancer medication combinations, the significant functions of Chinese herb therapies as adjuvant therapies for cancer treatment are described in detail.

## 1 Introduction

Malignant tumors substantially endanger human life and quality of life and place a heavy financial burden, particularly on developing nations. They are one of the main reasons for increased mortality globally and a key barrier to raising the average lifespan of people. According to the latest study on cancer released by the World Health Organization, data from 185 countries for the year 2022 show that there are about 20 million new cancer cases and nearly 9.7 million cancer deaths globally, and about 35 million cancer cases are predicted by 2050, an increase of 77% from 2022, and that the incidence of cancer and deaths from the disease is increasing rapidly worldwide (1). As a result, researching and creating efficient preventive and therapeutic programs to deal with cancer continues to be a major issue that the medical community worldwide will need to address in the future.

The current state of mainstream allopathic medicine’s treatment options for malignant tumors include radiation therapy, chemotherapy, targeted therapy, and immunotherapy. For patients with middle- and late-stage cancers, these treatments have demonstrated short-term effectiveness. At the same time, they can also produce toxic effects on normal cells during single or combined use. The resulting series of adverse reactions and toxic side effects (e.g., neuropathic cold pain, mechanical pain, and cardiotoxicity) have greatly limited their application and promotion. At the same time, they also have a serious negative impact on the quality of life of patients and even interrupt treatment (2). Therefore, effective management and mitigation of the toxic side effects of antitumor drugs are crucial to protect the physical and psychological health of patients.

Multidrug resistance (MDR) induced by the continuous application of chemotherapeutic agents is also considered to be one of the main reasons for the failure of clinical chemotherapy treatments. MDR leads to a decrease in the absorption and utilization of drugs by the body, which reduces therapeutic efficacy, delays disease conditions, and even leads to tumor recurrence or metastasis, which seriously affects patient survival and quality of life. Therefore, it is extremely urgent to develop and explore therapeutic options that can assist in ameliorating the adverse effects induced by antitumor therapy and improving MDR.

The current state of tumor treatment has moved into a more varied and comprehensive phase, and patients’ desires for longer survival times and better quality of life cannot be met by using conventional allopathic medicine alone. Complementary and alternative medicine (CAM), led by traditional medicine, has been chosen and recognized. Compared with mainstream allopathic medicine, CAM therapies are effective in improving patients’ quality of life, extending survival, reducing toxicity, increasing efficacy, and lowering the risk of recurrence and metastasis despite having a limited capacity to kill and eradicate tumor cells and unclear short-term efficacy.

Chinese herbal medicine, with its distinctive theoretical and treatment methods, forms the basis of traditional Chinese medicine, which is a significant component of CAM. Many studies have confirmed that Chinese herbs are more effective in assisting mainstream allopathic medicine (radiotherapy, chemotherapy, targeted therapy, and immunotherapy) in treating tumors to reduce toxicity and increase efficacy, and they can improve therapeutic efficacy in patients. Antitumor drugs tend to accumulate in specific anatomical regions, such as the cardiovascular system, kidneys, liver, or lungs, leading to cardiotoxicity, nephrotoxicity, hepatotoxicity, and pulmonary fibrosis (3–5). Numerous anticancer medications target DNA in proliferating cells, and they mostly cause side effects in tissues such as the gastrointestinal tract and bone marrow that strongly promote cell proliferation. These side effects can include immunosuppression, myelosuppression, and gastrointestinal toxicity (6).

Traditional natural medicines and Chinese patent medicine (CPM) are important components of Chinese herbs, and the monomer of Chinese herbs monomer (CHM) is the main active ingredient for traditional natural medicines to exert pharmacological effects. The good efficacy and safety of CHM and CPM as adjuvant therapies for treating tumors have been demonstrated by numerous studies. In this paper, we will review recent advances in laboratory and clinical studies on the use of CHM and CPM for enhancing antitumor efficacy and improving adverse effects during anticancer treatment. Clinical anticancer CHM and CPM, their synergistic effects with conventional allopathic medicine and its medications, and the combination schemes of innovative drug delivery systems from fundamental research and clinical trials, respectively, have all been chosen and discussed. This review advances the knowledge of TCM-assisted tumor treatment and offers insightful data and directions for the investigation and development of safer and more potent anticancer medications.

## 2 Chinese herb monomer

### 2.1 Ginsenosides

Ginseng is the dried root or rhizome of Panax ginseng C.A. Mey. It is one of the most valuable and widely used traditional Chinese medicines in China, and because of its remarkable medical efficacy, much progress has been made in understanding the active compounds and how they work. The antitumor effects of ginseng on a variety of malignant tumors, such as lung cancer, gastric cancer, and liver cancer, have been widely and thoroughly studied. Ginseng has a variety of antitumor-active components, and ginsenosides are the most important components of ginseng that exert antitumor effects.

In recent years, with research on modern traditional Chinese medicine pharmacology, the potential effects of ginsenosides, which exert powerful antitumor effects on a variety of cancer species by regulating multiple signaling pathways and genes, have gradually developed and improved. Ginsenosides, meanwhile, have become a popular drug in the adjuvant treatment of malignancies and offer a wide range of development prospects as supplementary and adjuvant medications to many contemporary anticancer agents and methods, which can synergistically increase their therapeutic effects and reduce toxicity. Various isoforms of ginsenosides (Rg1, Rg3, Rg5, Rb1, and Rh2) have synergistic effects with modern antitumor drugs (adriamycin and platinum).

The triple-negative breast cancer (TNBC) tumor is characterized by resistance to treatment, with adriamycin (DOX) commonly prescribed despite limitations in long-term use. Treatment of MDA-MB-231 cells with DOX following pretreatment with ginsenoside Rg1 (GRg1) resulted in a decrease in IC50, activation of DNA damage-responsive elements, and upregulation of apoptosis-associated genes (p21, TP53, Bax, and caspase-9/3), ultimately inducing apoptosis. GRg1 synergistically enhances the efficacy of DOX and has significant chemosensitizing effects (7).

Currently, among the many subtypes of ginsenosides, GRg3 has been the most intensively and extensively studied. A number of cancers have been treated with GRg3 and DOX combination therapy regimens. The mechanism of action of GRg3 in combination with DOX in osteosarcoma was investigated by Zeng et al. The results showed that GRg3 combined with DOX inhibited 143B cell proliferation, lung metastasis, and angiogenesis and improved DOX-induced weight loss and cardiotoxicity by regulating the mTOR/HIF-1α/VEGF and EMT pathways. Intervention with GRg3 improved the therapeutic efficacy of DOX and reduced its toxicity and side effects, which indicated the safety of DOX (8). One of the main characteristics of metabolic reprogramming in tumors is the generation of energy in a manner dependent on glycolysis. There is growing evidence that aerobic glycolysis is associated with tumor growth and chemotherapy resistance. Tamoxifen (TAM) resistance is a great challenge in breast cancer treatment. Studies have shown that the expression of the key glycolysis-related mediator PFKFB3 is upregulated in the TAM-pretreated drug-resistant cell lines MCF-7/TamR and T-47D/TamR and that intervention with GRg3 inhibits this expression and proliferation of drug-resistant cells and inhibits glycolysis and sensitization to TAM (9).

Zinc finger protein 91 (ZFP91) is an innovative E3 ubiquitin ligase that has been linked to drug resistance in cancers and has been demonstrated to be increased in a range of tumor types. ZFP91 overexpression is prevalent in pancreatic cancer and negatively correlates with overall survival. Gemcitabine (GEM) is a drug for the treatment of advanced pancreatic cancer and is the first-line standard anti-pancreatic cancer drug. The survival of pancreatic cancer patients is far from satisfactory due to the rapid development of drug resistance. Pan et al. explored the mechanism of action of GRg3 in combination with GEM in the treatment of pancreatic cancer by constructing a nude mouse transplant tumor model of PANC-1 tumors. The results showed that GRg3 was able to reduce the expression of ZFP91 in pancreatic cancer cells in a dose-dependent manner, improve ZFP91-induced tumor resistance and enhance the drug sensitivity and antitumor efficacy of GEM (10). When GRg3 and sorafenib were combined, they significantly reduced the growth and viability of the hepatocellular carcinoma cell lines HepG2 and Bel7404. Additionally, by controlling the PI3K/Akt signaling pathway and HK2-mediated glycolysis, GRg3 intervention increased the therapeutic effects of sorafenib and increased tumor cell sensitivity (11).

In gastric cancer cells, GRg3 combined with cisplatin modulates the Wnt/β-catenin signaling pathway, downregulates the expression of pathway-associated proteins (Wnt, β-catenin, C-myc, and cyclin D1) and EMT-associated proteins, significantly inhibits the activity of SGC-7901 cells, increases cisplatin-induced apoptosis, enhances the sensitivity of gastric cancer cells to cisplatin chemotherapy, enhances the efficacy of chemotherapy, and inhibits the malignant progression of tumors (12). Furthermore, it has been demonstrated that SOX2 is a target gene of miR-429 in gastric cancer cells and that miR-429 is connected to cisplatin resistance in cisplatin-resistant gastric cancer cells. After GRg3 treatment, the expression of apoptosis-related genes, which target SOX2 and the PI3K/AKT/mTOR signaling pathway, was upregulated in gastric cancer cells, cell migration was significantly inhibited, GRg3 alleviated the drug resistance of gastric cancer cells, and sensitivity to cisplatin was enhanced (13).

The expression of ATP-binding cassette (ABC) transporter proteins, which are ATP-dependent efflux pumps, is generally thought to be the cause of multidrug resistance in tumor cells. The expression of the ABCB1 gene is negatively correlated with the effectiveness of first-line chemotherapy, and high expression of the ABCB1 gene predicts a poor prognosis for patients with advanced disease (14). The Hedgehog signaling pathway is a classical signaling pathway that controls the development of embryos, and the infinite expansion of tumor cells is also a process of uncontrolled growth and proliferation of tumor cells as members of the Hedgehog signaling pathway, mutations, expression changes, and methylations. SHH, PTCH1, and GLI can cause many potential changes. It has been shown that in NSCLC, GRb1 can decrease the expression levels of ABCB1, SHH, PTCH1, GLI2, and the apoptosis-resistant protein Bcl-2 and upregulate the expression of the cellular pro-apoptotic protein Bax, which advances the pro-apoptotic process of cisplatin. GRb1 increased the intracellular drug concentration of cisplatin by targeting ABCB1 and the Hedgehog pathway, reversing cisplatin resistance in A549/DDP cells and improving its efficacy (15). In nasopharyngeal carcinoma cells, Li et al. reported that GRb1 combined with apatinib could inhibit tumor cell glycolysis, reduce tumor cell proliferation and migration, upregulate the immune-related indices CD3^+^ and CD4^+^, increase apoptosis, enhance the expression of immune regulatory molecules, and exert synergistic anticancer effects on nasopharyngeal carcinoma cells via ginsenosides to inhibit the malignant progression of nasopharyngeal carcinoma (16). In cervical cancer chemotherapy, paclitaxel (PTX) resistance has become a major challenge and therapeutic difficulty. Ramesh et al. applied GRg5 to explore the mechanism of reversing PTX resistance. The findings demonstrated that by controlling two signaling pathways, Akt and NF-κB, and related apoptotic proteins (Bax, caspase-9/-3, Bcl2, Bcl-XL, c-IAP-1, and MCL-1), GRg5 increased the apoptotic process, enhanced the chemosensitivity of HeLa cells to PTX and reversed the chemoresistance of PTX (17).

In lung cancer cells, the use of everolimus in combination with GRh2 (Eve-Rh2), by upregulating c-MYC expression, caused the mediated accumulation of tribble homologous protein 3 (TRIB3)/P62+ aggregates that triggered the apoptosis process and inhibited the malignant progression of lung cancer cells, providing a new synergistic therapeutic strategy (18). Sunitinib was developed with the primary goal of being used for the treatment of clear cell renal carcinoma (ccRCC) and was able to increase the progression-free survival of patients with ccRCC effectively. The mechanism by which GRh2 synergizes with sunitinib to cure ccRCC was investigated by Hwang et al. The results showed that combined treatment with GRh2 and sunitinib enhanced G2/M blockade and DNA damage enhancement (upregulation of 8-OHdG expression), demonstrated inhibitory effects on ccRCC cell viability, and was validated *in vivo*. In combination with sunitinib, GRh2 provides a new therapeutic option for patients with ccRCC (19).

Pancreatic cancer is a cold tumor, an immune cell-suppressive tumor with no or few immune cells (dendritic cells and T cells) in the tumor tissue, and immunotherapy is not effective. Given that GRh2 possesses immune-promoting properties, it might be worthwhile to investigate the use of GRh2 in combination with GEM to treat pancreatic cancer. Relevant studies have shown that GRh2 combined with GEM (GEM-Rh2) affects pancreatic cancer cells and activated dendritic cells (DCs) by regulating the BCL10-MALT1/NF-κB pathway, with a significant increase in the number of DCs. The key target of DC function enhancement, central articulation protein caspase recruitment domain-containing protein 9, a key target of DC function enhancement, was significantly upregulated, and GRh2 enhanced the efficacy of GEM, providing a new therapeutic strategy and option for the “hot-cold” transformation of tumors (20).

Radiotherapy is widely used in the radical, adjuvant, and palliative treatment of tumors. Although the tumor control rate has grown dramatically due to advancements in radiation technology, this method is still severely constrained by the harmful side effects of radiation. Studies have shown that GRh2 can inhibit radiotherapy-induced NF-κB activity and PD-1 receptor expression by suppressing the MAPK pathway in a CT26/luc mouse colon cancer cell model, and GRh2 combined with radiotherapy treatment significantly slowed tumor growth and overall survival, inhibited tumor progression, and improved therapeutic effects (21).

Radiotherapy resistance decreases the therapeutic efficacy of radiation therapy, while radiotherapy toxicity limits its therapeutic duration. In nasopharyngeal carcinoma cells, Hu et al. explored the sensitizing therapeutic effect of GRg3 combined with radiotherapy. The results showed that GRg3 could block radiotherapy-induced EMT, reduce the nuclear translocation of EGFR and the expression of DNA-dependent protein kinases, significantly promote the apoptosis of HNE1 and CNE2 cells; thus, GRg3 could be further promoted as a potential radiotherapy sensitizer (22). In lung cancer cells, GRg3 and GRg5 also have radiosensitizing effects. GRg3 ultimately achieves the therapeutic effect of radiotherapy sensitization by inhibiting the proliferation of lung cancer cells, accelerating the process of apoptosis, increasing the proportion of cells in the G2/M phase, preventing the formation of cell colonies, and decreasing the expression levels of PI3K, p-AKT, and PDK1 in cells (23). The effects of GRg5 on lung adenocarcinoma sensitization to radiotherapy were investigated by Bai et al. The results showed that in A549 and Calu-3 cells, GRg5 combined with radiotherapy-induced cell cycle arrest in the G1 phase and advanced apoptotic processes, upregulated p62, SRC, CDK4, RAF1, and ULK1 protein expression, inhibited LC3 expression; and enhanced radiotherapy-derived DNA damage, suggesting that ginsenosides are sensitizing and adjuvant agents for radiotherapy (24).

GRb1, which has good solubility and antitumor effects, has attracted increased amounts of attention. Lu et al. studied and explored the self-assembling behavior of Rb1 in the treatment of breast cancer by applying nanotechnology to prepare GRb1 nanocapsules wrapped with protopanaxadiol (PPD) and PTX to form coloaded nanoparticles (GPP-NPs). The synthesized GPP-NPs were stable and released PTX and PPD in a sustained manner. Compared with the *in vivo* anticancer impact of PTX injection, the experimental results demonstrated much stronger tumor inhibition (64.95% *vs*. 43.17%) and superior tumor targeting of GPP-NPs (25).

By allowing hydrophilic polymeric chains to accommodate aqueous biofluids, nanohydrogels exhibit high water absorption, minimal toxicity, and good biocompatibility. For some hydrogels, once stimulated by stimuli such as changes in temperature, pH, and ionic strength, a phase transition occurs, and changes such as changes in porosity and hydrophilicity allow for the controlled release of drugs. Cui et al. prepared nanohydrogels loaded with lapatinib and GRg1 (OCMC-g-Suc-β-CD) using β-cyclodextrin (β-CD) and amino acids as the functionalization moieties and performed *in vitro* experimental validation for the treatment of lung cancer. The produced nanohydrogels showed high cytotoxicity against lung cancer cells, according to the experimental data (A549). In a zebrafish model, OCMC-g-Suc-β-CD significantly inhibited the expression of the EGFP-Kras v12 oncogene in the liver, showing a more desirable anticancer effect (26). P-glycoprotein (P-gp) is a typical class of multidrug resistance transporter protein. P-glycoprotein-mediated active transport, which transports antitumor drugs out of the cell, reduces the accumulation of drugs in the cell, leading to a reduction in drug efficacy and drug resistance and, ultimately, drug resistance. Therefore, the development of clinically effective P-gp inhibitors is highly important. Long et al. combined GRh2 and PTX and constructed a nanometer temperature-sensitive gel drug delivery system (SLNs-Gel) to achieve continuous release of GRh2 and PTX in the tumor microenvironment, effectively combating multidrug resistance in breast cancer. The outcomes demonstrated that SLNs-Gel improved the medication sensitivity of PTX while also dramatically reducing the growth of tumor cells. SLNs-Gel showed great potential in combating drug-resistant breast cancer cells (27). Currently, chemotherapy combined with immune checkpoint inhibitors is a clinically effective antitumor regimen. In the treatment of TNBC, Wu et al. combined GRg3 with DOX encapsulation to construct temperature-sensitive hydrogels (Rg3-DOX-PNPs) and combined them with PD-L1 to observe their antitumor effects. According to these findings, the combination of the hydrogel and PD-L1 increased memory T-cell recruitment and decreased adaptive PD-L1 enrichment. Additionally, the novel system demonstrated strong anti-breast tumor effects, offering a novel approach to the “cold-hot” transition of breast cancer (28).

Lipid nanodelivery systems have the advantages of prolonging the retention time of active ingredients in traditional Chinese medicine *in vivo*, enhancing targeting, and increasing solubility. GRh2 liposomes (LTL@Rh2@Lipo-GE11), which target EGFR, were able to counteract breast cancer cells with high EGFR hyperexpression more accurately, showed high specificity and strong cytotoxicity, inhibited the growth and migration of breast cancer cells, and are a promising therapeutic option (29). Similarly, liposomes containing GRg3 also possessed certain anti-TNBC effects. Gu et al. used GRg3 as a membrane material and combined it with artemisinin and PTX to establish liposomes. According to these findings, liposomes containing GRg3 could more significantly inhibit the proliferation and migration of breast cancer cells after 24 hours of administration than free drug or DOX. Additionally, the toxicity of the drug toward normal cells was lower. These findings suggest a direction for the development of traditional Chinese medicine nanomolecule drugs with decreased toxicity and increased therapeutic effects (30).

Sonodynamic therapy (SDT) is an emerging method of using ultrasound combined with acoustic sensitizers to improve the efficiency of tumor treatment and can significantly enhance the therapeutic effect of photodynamic therapy, chemotherapy, and immunotherapy on tumors. The generation and release of large amounts of ROS is one of the effective mechanisms of sonodynamic therapy. The tumor microenvironment is a hypoxic environment that is not able to generate enough ROS to achieve good therapeutic effects. Therefore, it is urgent to explore acoustic sensitizers that can effectively increase the level of intracellular ROS to improve antitumor effects. Titanium dioxide (MHT) is a photosensitizer, and Yang et al. combined and loaded GRk1 and MHT onto a novel manganese-doped acoustic sensitizer, Rk1@MHT-based SDT, to enhance the therapeutic effect on tumors. The results showed that GRk1 inhibited the glutathione pathway and enhanced intracellular ROS levels by blocking glutaminase. Moreover, manganese doping led to a significant increase in the absorption of MHT, which increased the ROS content under ultrasonication. The investigation of Rk1@MHT-based SDT offers a novel approach for developing a cutting-edge, noninvasive tumor treatment method (31).

Normal human tissues and blood have a pH of approximately 7.4, whereas the extracellular environment of tumor tissues has a slightly acidic pH of 6.5–7.2. In tumor cells, the pH of lysosomes and endosomes is further reduced (pH 4.0–6.0), and this microacidic environment provides a new strategy for nanocarrier development. pH-responsive prodrug delivery systems, as nanodelivery systems, can achieve passive or active targeting and reversal of multidrug resistance in tumors through the low pH of tumor tissues, which can greatly improve the efficacy of antitumor drugs. In addition to being extensively utilized in anticancer therapy, DOX has serious adverse effects. Rahimi et al. constructed a novel drug delivery system (GO-Rg3-DOX) using GRg3 combined with graphene oxide (GO) for nanocarrier construction, added DOX, and applied it to treat Huh7 hepatoma cells and MDA-MB-231 breast cancer cells. The results showed that the combination of GRg3 and GO significantly reduced the toxicity of GO, downregulated the expression of transcriptional regulatory genes, upregulated the expression of apoptotic genes, decreased tumor cell viability, inhibited GO-induced tumor cell proliferation, and ameliorated DOX-induced cytotoxicity. Its potential applications in the treatment of breast cancer and hepatocellular carcinoma are extensive (32).

Patients with advanced metastatic colorectal cancer are commonly treated with oxaliplatin, a third-generation platinum-based chemotherapeutic drug. During oxaliplatin treatment, 85–95% of patients experience rapid onset of neuropathic pain without motor impairment, peaking 24–48 hours after starting the medication (33) and lasting for approximately 1 week. Tricyclic antidepressants, anticonvulsants, opioids, and nonsteroidal analgesic and anti-inflammatory drugs are currently used to treat chemotherapy-induced neuropathic pain. However, the application of more effective and safer drugs for pain prevention is often more important than treatment. Therefore, the search for new, safe, and effective alternative drugs for the prevention of oxaliplatin-induced neuropathic cold and mechanical pain is of great clinical importance. Park et al. reported that the Spinal Noradrenergic System plays an important role in the prevention of neuropathic pain (34). A frequent treatment for patients with advanced metastatic colorectal cancer is oxaliplatin, a third-generation platinum-based chemotherapy medication. Eighty-five to 95% of patients receiving oxaliplatin suffer a fast onset of neuropathic pain without motor impairment, which peaks 24 to 48 hours after the medicine is started.

The cardiotoxicity of antitumor drugs is a common adverse reaction, mostly caused by anthracyclines, such as DOX and Zoerythromycin. Studies have shown (35) that in a mouse model of DOX-induced breast cancer cardiotoxicity, GRh2 can mitigate the cardiotoxicity induced by chemotherapeutic drugs and reduce the toxic side effects by attenuating cardiac rational remodeling caused by myofibroblastic transformation and endothelial-mesenchymal transition, regulating the T-cell activation and cytokine response induced by DOX, and promoting the onset of cellular apoptosis. Trastuzumab also has high cardiotoxicity and has the potential to induce left ventricular dysfunction. In this type of cardiotoxicity in rats generated by trastuzumab (36), the clonogenic ability of primary human cardiomyocytes was reduced, apoptosis-related proteins were upregulated, and apoptosis was promoted; moreover, GRg2 intervention improved the above situation, restoring cell clonogenicity and resistance to apoptosis. In the field of chemotherapeutic drug-related cardiotoxicity prevention and treatment, the application and development of GRh2 and GRg2 have great potential.

### 2.2 Atractylenolide

The protagonist Atractylodes rhizome is derived from the dehydrated rhizome of Atractylodes macrocephala Koidz in the Asteraceae family. The active ingredients of Atractylodes rhizome exhibit extensive antitumor activity against breast cancer, prostate cancer, colorectal cancer, and other tumor cells, and the active ingredients of Atractylodes rhizome include lactones, polysaccharides, and volatile oils. Among them, the antitumor activity of atractylenolides I, II, and III and polysaccharides from Atractylodes macrocephala koidz is the most significant, and atractylenolide I (ALT-1) is the most widely studied.

Prostate cancer is the second most common malignant tumor worldwide and the fifth leading cause of cancer deaths in men (1). Cabozantinib is a drug commonly used to treat thyroid or kidney cancer, and a recent study revealed that this drug is effective at preventing the proliferation of tumor cells in invasive prostate cancer in mice (37). Some clinical studies have shown that cabozantinib can activate the neutrophil-mediated antitumor innate immune system to exert antitumor effects. HSP27, a member of the low-molecular-weight heat shock protein family, is an important protein involved in drug resistance, cell growth, apoptosis, tumorigenesis, and metastasis, and high expression of Hsp27 in tumor cells is correlated with chemotherapy resistance. Studies have shown that ATL-1 inhibits the proliferation of DU145 and PC-3 cells and enhances the drug sensitivity of prostate cells to cabozantinib by modulating the Hsp27/eIF4E signaling pathway, decreasing cell viability and upregulating the expression of proapoptotic proteins (caspase-3, PARP, and Bax.) ATL-1 has broad applications as a drug sensitizer for cabozantinib (38). First, connective tissue growth factor (CTGF) was shown to stimulate fibroblast proliferation by acting on fibroblasts in a chemotactic and mitogenic manner. CTGF also affects cell proliferation, migration, and differentiation in different cell types. CAFs and their secreted factors are closely related to the development of chemoresistance. Studies have shown that ATL-1 can inhibit the migration of TNBC cells and the ability of fibroblasts to transform into CAFs by downregulating the expression of CTGF in TNBC cells and fibroblasts, preventing TNBC cells from metastasizing to other regions and the formation of chemoresistance mediated by CAFs. By affecting CTGF and CAFs, ATL-1 prevents tumor metastasis and drug resistance, improves TNBC-induced chemoresistance, enhances the therapeutic efficacy of PTX, and provides a new solution for effectively prolonging the survival of TNBC patients (39). Chemotherapeutic drugs such as 5-fluorouracil (5-FU), cisplatin, mitomycin, and DOX are frequently administered to patients with colorectal cancer. X inactivation-specific transcript (XIST) is specifically and highly expressed in a variety of tumors and is able to promote tumor cell proliferation, migration, and invasion *in vitro*. ROR1 is a transmembrane receptor tyrosine kinase that regulates cell division, proliferation, migration, and chemotaxis. ROR1 expression is significantly elevated in a variety of hematological cancers and solid tumors and has been associated with epithelial–mesenchymal transition (EMT), metastasis, and invasive disease. Studies have shown that ATL-2 can inhibit the viability and proliferation of colon cancer cells by regulating the expression of various genes in the XIST/miR-30a-5p/ROR1 pathway and, at the same time, enhance the chemosensitivity of colon cancer cells and increase the antitumor efficacy of chemotherapeutic agents. ATL-2 can be used as a sensitizer and complementary agent to chemotherapeutic agents in collaborative chemotherapeutic regimens (40).

Approximately 40–80% of patients with advanced tumors will have cancer cachexia, and approximately 20% of patients with tumors can die of cancer cachexia directly. Thus, investigating the possible mechanisms of action of natural medicines against cachexia and developing efficient anticachexia medications are imperative. Fan et al. constructed a C26-homozygous BALB/c mouse model and explored the mechanism by which ATL-1 alleviates malignant disease in tumors. The results showed that ATL-1 could reduce the production of extracellular vesicles (EVs) and IL-6 secretion by regulating the STAT3/PKM2/SNAP23 pathway, which inhibited aerobic glycolysis and suppressed the ability of tumor cells to induce muscle atrophy, thus alleviating malignant disease. ATL-1 inhibited malignant disease of tumor origin and improved survival (41). Bleomycin is a multicomponent complex antibiotic composed of alkaline glycopeptides produced by Streptomyces verticillioides that has antitumor effects. One of its toxic side effects is pulmonary fibrosis, which is irreversible damage that seriously affects the quality of life of patients. Research has been conducted on the mode of action of ATL-3 in reducing bleomycin-induced prostate cancer. The results showed that ATL-3 was able to reverse the effects of Nrf2 interference on lung fibrosis injury, ameliorate bleomycin-induced pulmonary fibrosis and oxidative stress, and enhance survival by modulating the Nrf2/NQO1/HO-1 signaling pathway and decreasing the expression of apoptosis-related proteins (caspase-3/9), TGF-β, α-SMA, and inflammatory factors (42).

### 2.3 Astragaloside

Astragaloside, a major saponin compound extracted from the root of the traditional Chinese herb Astragalus membranaceus, is a key component of the pharmacological effects of Astragalus membranaceus and is currently widely used to treat tumors.

Improving the sensitivity of cells to anticancer drugs is a crucial aspect of treatment that requires further study. Astragaloside combined with antitumor drugs can significantly reduce drug resistance, with remarkable efficacy, and is a widely promising anticancer component of Chinese herbs (43). Dai et al. reported that astragaloside IV could inhibit the proliferation of NSCLC cells, while astragaloside IV combined with chemotherapy could increase the sensitivity of tumor cells to gefitinib by regulating the level of SIRT6 in NSCLC cells (44). Wang et al. found that Astragaloside exposed NSCLC to anlotinib by modulating the inhibition of the miR-181a-3p/UPR-ERAD axis, thus overcoming the resistance of NSCLC to anlotinib and revealing a very promising treatment for antitumor drug resistance (45). Li et al. explored whether astragaloside could enhance the sensitivity of lung adenocarcinoma cells to bevacizumab, and the results showed that astragaloside IV inhibited the increase in cell viability and the expression of proapoptotic proteins in A549 cells and, at the same time, reversed the effects of bevacizumab on autophagy-related proteins and the levels of phosphorylated AKT and mTOR. By blocking the autophagy pathway, astragaloside IV amplifies the effect of bevacizumab in suppressing the proliferative viability and encouraging the death of A549 cells (46). Lai et al. reported that astragaloside IV increased the sensitivity of NSCLC cells to cisplatin by inhibiting the viability of A549 and H1299 cells, increasing the rate of apoptosis, and inhibiting endoplasmic reticulum stress and cellular autophagy, providing a new therapeutic strategy for the combination of cisplatin with astragaloside IV for the treatment of cisplatin resistance (47). He et al. reported that astragaloside IV enhanced the sensitivity of prostate cancer cells to carboplatin by inhibiting the AKT/NF-κB signaling pathway, thus inhibiting carboplatin-induced epithelial-mesenchymal transition, providing a new mechanism by which astragaloside IV overcomes platinum-based drug chemoresistance (48). According to Huang et al., astragaloside IV significantly reduces the stemness of breast cancer cells, decreasing the resistance of MCF7-CSCs to PTX and increasing the therapeutic efficacy of PTX (49). Zheng et al. reported that astragaloside IV increased the chemosensitivity of breast cancer to PTX by inhibiting the expression of follicle protein-1, which in turn activated the eNOS/NO/ONOO pathway as a target for reversing tumor resistance (50). Qu et al. reported that astragaloside IV enhanced the sensitivity to cisplatin chemotherapy by increasing tumor cell apoptosis, slowing tumor growth and inhibiting the expression of multidrug resistance-associated proteins in tumor cells from enhancing the antitumor effect of cisplatin on hepatocellular carcinoma, which provided new insights into the combined use of chemotherapeutic agents and natural ingredients in the treatment of cancer (51).

Nanomedicines have proven to be advantageous in the field of tumor therapy, offering more accurate and potent methods for treating tumors. Xu et al. prepared a novel multifunctional nanomedicine (ursolic acid/astragaloside IV@PDA-HA) modified with hyaluronic acid in high yield and low cost that combines chemotherapy, photothermal therapy and immunotherapy with proactive tumor-targeting ability. The nanodelivery system significantly improved ursolic acid-mediated cytotoxicity and antimetastatic ability against NSCLC cells. Furthermore, Astragaloside IV enhanced the autoimmune response to antigens associated with tumors, hence impeding the proliferation and distant metastasis of NSCLC cells and considerably reducing tumor growth. The application of the nanotechnology Astragaloside not only significantly clears primary tumors of NSCLC *in vitro* and *in vivo* but also significantly inhibits distant metastasis *in vitro* and *in vivo*, which has great potential for development (52). Yue et al. prepared a novel liposome-targeted codelivery system comodified with a folate ligand (FA) and an octa-arginine polypeptide (R8), which effectively improved the tumor-targeting and cellular uptake of liposomes and significantly inhibited the proliferation of MDA-MB-231/DOX-resistant cell lines, and the FA-R8-LPs significantly inhibited MDA-MB-231/DOX cell-inoculated tumor growth and overcame DOX resistance in a nude mouse tumor model, showing good antitumor effects. The combination of medications is crucial for treating TNBC, and the use of astragaloside IV, which is based on an FA and R8 dual-modified liposome-targeted codelivery system, offers a new and successful approach (53).

DOX is a highly effective broad-spectrum antitumor drug with remarkable antitumor efficacy but also has serious cytotoxicity. After DOX enters cardiomyocytes, it can promote the production of ROS and malondialdehyde (MDA) and reduce the activities of superoxide dismutase (SOD) and glutathione peroxidase (GPX), thus reducing the levels of GPX and SOD in the myocardium and leading to oxidative stress injury, cardiotoxicity, and cardiomyocyte injury (54, 55). According to Lin et al., astragaloside IV treatment effectively ameliorated cardiomyocyte injury and apoptosis and was a potential therapeutic strategy to lessen the side effects of antitumor drugs. It also attenuated DOX-induced weight loss, myocardial injury, cardiomyocyte apoptosis, myocardial fibrosis, and cardiac dysfunction (56).

### 2.4 Polyphyllin

Paris polyphylla is an herb that is part of the Liliaceae family and genus Serpentine. It has been studied and used extensively in the field of therapeutic herbs. In recent decades, the pharmacological activities and possible potential mechanisms of action of Paris polyphylla and its active ingredients have been identified, and its antitumor effects are one of its main pharmacological effects. Polyphyllin (PP), the most important component of Paris polyphylla, exerts antitumor effects and is widely used clinically for the treatment of various cancers. In recent years, with the in-depth excavation and exploration of Chinese herb components in modern pharmacology, the potential antitumor effects of an increasing number of Chinese herb-active ingredients have been gradually revealed, and these compounds have begun to work synergistically with modern antitumor regimens, with the aim of achieving more efficient and safer therapeutic regimens and prolonging the pain-free survival of patients. Different PP subtypes (I, II, VI, and VII) work in concert with contemporary anticancer medications (ositinib, DOX, and platinum).

Patients with advanced non-small cell lung cancer who present with the T790M mutation may have a better chance of surviving if they take ocitinib, a very potent and selective EGFR mutant inhibitor. However, ocitinib resistance has proven to be a barrier to effective lung cancer treatment. The mechanism of action of PPI in reversing ocitinib resistance was explored by Lai et al. The results of *in vitro* and *in vivo* experiments showed that PPI could reverse ositinib-induced NSCLC resistance and enhance the therapeutic effect of ositinib by regulating the PI3K/Akt signaling pathway, modulating apoptosis-related proteins and advancing the process of apoptosis (57). Platinum is a commonly used chemotherapeutic agent for the treatment of NSCLC, and the reversal of chemoresistance is a common challenge. Peng et al. developed an A549 cisplatin resistance model to investigate the synergistic effect of PPII and cisplatin. These findings demonstrated that PPII and cisplatin might increase the expression of proapoptotic proteins, inhibit the cell cycle in the S and G2/M phases, and accelerate the process of mitochondrial apoptosis. PPII reversed cisplatin-induced resistance in lung cancer cells and enhanced the efficacy of cisplatin against lung cancer, which further promoted the synergistic effect of the active ingredients of Chinese herbs with synergistic chemotherapeutic agents (58). The cancerous inhibitor of protein phosphatase 2A (CIP2A) is a human oncoprotein. Several studies have shown that CIP2A is widely and highly expressed in a variety of human malignant tumors and plays an important role in maintaining the malignant phenotype of cells and promoting cell proliferation and malignant transformation. Feng et al. treated cisplatin-resistant NSCLC with PP I and VII. The results showed that PP I and VII could inhibit the proliferation and migration of A549-resistant cells and enhance the sensitivity of resistant cells to cisplatin by regulating the CIP2A/AKT/mTOR signaling pathway and upregulating p53, which causes apoptosis and enhances the effect of chemotherapeutic therapy. PP I and VII have broad prospects for the development of cisplatin as chemotherapeutic sensitizers (59, 60).

Approximately 40–50% of intracranial tumors are gliomas, which are the most prevalent primary brain tumors. It has a poor therapeutic effect, is difficult to cure, easily recurs, rapidly progresses, and has a poor prognosis. Temozolomide is a new type of oral alkylating agent with broad-spectrum antitumor activity that can pass through the blood-brain barrier and has a bioavailability close to 100%, and it is the first-line drug for the treatment of glioma internationally. The primary cause of chemotherapy failure and the barrier to glioma treatment efficacy is drug resistance. Feng et al. showed that PPI promoted ROS-induced apoptosis and autophagy and inhibited tumor cell proliferation in a concentration-dependent manner by regulating the MAPK and NRF2 signaling pathways (61). In addition, it has been found (62) that PP VII inhibited the activation of the AKT/mTORC1 pathway by upregulating the ROS content, upregulating the expression of the apoptotic proteins Bcl2 and Bax, and significantly inhibiting the cellular activity of U87-MG and U251 cells. The therapeutic strategy of combining PP I and VII with temozolomide enhanced the effect of temozolomide, reversed drug resistance in patients with gliomas, and effectively prolonged glioma-resistant patient survival, which has broad application prospects and practical significance. DOX is a common medication used to treat colorectal cancer. Studies have shown that the combination of PP ethanol extracts (3-o-β-chacotrioside and PP VI) with DOX can induce autophagy in colorectal cancer cells by upregulating autophagy markers, resulting in significant antitumor effects. The combination of 3-o-β-chacotrioside and PP VI in combination with DOX enhances the therapeutic effect of DOX, which is worthy of further exploration as a new adjuvant chemotherapeutic regimen (63). The immunotherapeutic strategy of “cold-hot” transformation of malignant tumors has been widely used, and Xu et al. constructed a biomimetic nanosystem (RBC@Mn-MOF/PPI) based on a metal-organic framework wrapped with PPI. By triggering the release of PPI and Mn^2+^ and activating immune cell recruitment in the tumor microenvironment, the system remodels the tumor microenvironment, enhances the antitumor immune response mechanism mediated by the cGAS/STING pathway, and transforms malignant tumors from cold to hot, thus perfecting the mechanisms and therapeutic strategies related to antitumor immunotherapy (64).

### 2.5 Berberine

Berberine is a major quaternary ammonium compound extracted from the rhizome of Coptis chinensis Franch, a traditional Chinese herb in the family Trichoderma, and is a key component in the active action of Coptis chinensis Franch. The antitumor mechanism of berberine has been widely studied *in vitro* and *in vivo*.

Qu et al. reported that berberine enhanced the sensitivity of glioblastoma to temozolomide in an ERK1/2 signaling pathway transduction-related manner after treatment with the chemotherapeutic drug temozolomide and induced autophagy and apoptosis in temozolomide-resistant cells to improve antitumor drug resistance (65). Qian et al. found that berberine significantly downregulated the expression of P-gp/ABCB1 and MRP1/ABCC1 in a xenograft model of drug-resistant cell lines, which reduced the efflux of DOX, increased the uptake of DOX by tumor tissues and increased the concentration and retention of DOX in tumor cells; these findings provide practical and feasible new ideas for tumor therapy (66). By establishing GEM-resistant pancreatic ductal adenocarcinoma cells, Okuno et al. found that the application of berberine-induced pancreatic ductal adenocarcinoma cell blockage and apoptosis and reversed the resistance of pancreatic ductal adenocarcinoma to GEM by regulating the Rap1/PI3K-Akt signaling pathway (67). Chen et al. reported that berberine in combination with ositinib synergistically and selectively reduced the survival of ositinib-resistant EGFR-mutant NSCLC cell lines and enhanced the growth inhibition of met-amplified ositinib-resistant transplanted tumors in nude mice. To combat acquired resistance to ositinib caused by MET amplification, berberine may function as a naturally occurring MET inhibitor and work in concert with ositinib (68). Jabbarzadeh et al. concluded from drug transcriptomic analysis that Lapatinib and Berberine-treated drug-resistant TNBC cells showed upregulation of the PI3K/Akt signaling pathway under cytotoxic stress, which reduced antitumor drug resistance and improved therapeutic efficacy. Chen et al. reported that berberine combined with ectinib overcame ectinib resistance in NSCLC cells, led to intracellular ROS accumulation, induced autophagic cell death and apoptosis, significantly inhibited EGFR protein expression and activity, and was involved in the inhibition of cell migration and invasion (69). Aleissa et al. reported that the free energy of binding (ΔG) of berberine to ovarian cancer cell lines was -7.5 kcal/mol, and the free energy of binding of berberine to PALB/BRCA2 was -8.8 kcal/mol, which demonstrated that the effective and tight binding of the complexes enhances the radiosensitivity of ovarian cancer cell lines and is a promising nutritional candidate for anticancer diagnosis (70). Pan et al. discovered that while ER-α36 expression was inhibited in drug-resistant breast cancer cell lines, berberine effectively increased tamoxifen therapeutic sensitivity. These findings provide experimental support for the use of berberine as an adjuvant to tamoxifen therapy in tumor therapy (71).

Gao et al. assessed the potential effects of berberine on GEM-induced cytotoxicity in bladder cancer. Berberine attenuated GEM-induced activation of Akt and Rad51 through modulation of the PI3K/Akt pathway. Berberine enhances GEM-induced cytotoxicity, apoptosis, and migration in bladder cancer cells and synergistically enhances the antitumor effects of GEM (72). The therapeutic medication bleomycin (bleomycin) is commonly used to treat cancers such as lymphoma, melanoma, and lung cancer. As a side effect of bleomycin, pulmonary fibrosis occurs most frequently. Bleomycin injection causes damage and death of alveolar epithelial cells and fibroblasts, accelerates the release and production of collagen, and ultimately leads to pulmonary fibrosis. Ahmedy et al. reported that berberine significantly ameliorated bleomycin-induced pulmonary fibrotic changes by upregulating A2aR expression, inhibiting the SDF-1/CXCR4-related pathway, and suppressing inflammation and oxidative stress (73). Kim JH et al. reported that berberine exerted a protective effect against cisplatin-induced ototoxicity by decreasing the ROS content in cells, decreasing DNA fragmentation, and reducing mitochondrial stress (74). Berberine inhibited bacterial β-glucuronidase, attenuated irinotecan-induced loss of mucosal structure, and improved mucosal barrier function, all of which improved irinotecan-induced intestinal mucositis without compromising irinotecan’s efficacy against colorectal cancer (75).

By targeting and selectively accumulating in tumor tissues, nanoparticles enhance drug bioavailability and efficacy in a novel treatment approach called nanoparticle-targeted cancer therapy. CHM-Nanoparticle targeted cancer therapy is a more precise, effective, and safe treatment modality, providing better treatment options for cancer patients. Pearl et al. developed a novel nanocomposite combining the advantageous properties of erythrocyte membranes, the photoresponsive properties of gold nanoclusters (AuNCs), and berberine, which enhances the generation of reactive oxygen species and adaptability to hypoxic conditions, overcome the limitations of photodynamic therapy, and is a promising nanotherapeutic agent with significant potential in the treatment of advanced cancers (76). A berberine-C60 fullerene nanocomplex was used by Horak et al. to effectively inhibit Lewis lung cancer cell invasion *in vitro* and metastasis *in vivo*. It also demonstrated significantly greater efficiency in downregulating the levels of the transcription factors SNAI1, ZEB1, and TWIST1, deregulating the expression of the epithelial marker E-cadherin, and inhibiting the expression of tumor stem cell-like markers (77). Malyla V et al. encapsulated berberine in liquid crystal nanoparticles to improve its physicochemical function, targeted the β-catenin pathway to attenuate β-catenin expression, and inhibited the survival of human lung adenocarcinoma cell lines (78). Uma et al. prepared liposomes encapsulated with hyaluronic acid and chitosan, and berberine and DOX were encapsulated to enhance the inhibitory effect of the drug on lung cancer cells with good penetration ability and high cytotoxicity, with an IC50 value of 89.19 μg/300 μl, effectively targeting and entering into A549 cells to enhance cytotoxicity against A549 cells (79). To increase the anti-proliferative and anti-migratory effects of the medication with increased cytotoxicity and reduced migration, Xu et al. produced liposomes modified with glycyrrhizic acid for the codelivery of berberine and DOX. In addition, it significantly reduced the deposition of extracellular matrix and inhibited tumor angiogenesis, thereby suppressing tumor growth and showing excellent antitumor effects *in vitro* and *in vivo*, demonstrating its potential as an effective therapeutic strategy for hepatocellular carcinoma (80).

### 2.6 Poria cocos pachymaran

Poria cocos are rich in a variety of active ingredients, including triterpenes, polysaccharides, sterols, volatile oils, proteins, amino acids, and trace elements. Among them, pachymaran is the main active antitumor ingredient extracted from Poria cocos, and its antitumor mechanism of action has been widely studied both *in vitro* and *in vivo*.

An essential tactic in tumor therapy is synergistic and reducing toxicity, which aims to increase therapeutic efficacy while lowering harmful side effects in patients, making tumor therapy safer and more effective while also providing patients with better therapeutic options. Amini et al. reported that pachymaran can inhibit ovarian cancer cell proliferation, attenuate the cytotoxicity of chemotherapeutic drugs to NIH3T3 normal cells, and induce cancer cell apoptosis. Pachymaran combined with PTX can reduce the toxicity of chemotherapeutic drugs while improving the efficacy of drug-resistant ovarian cancer treatment by restoring p53 and mitochondrial apoptotic cells (81). Yin et al. investigated whether a kind of pachymaran with multiple biological activities and prebiotics can restore the homeostasis of the intestinal microenvironment and commensal flora, alleviate the adverse effects of the chemotherapeutic drug 5-FU and improve its therapeutic effect at the same time in ApcMin/+ mice, and the results showed that pachymaran could alleviate the side effects of 5-FU, such as weight loss and polyp burden, regulate intestinal inflammation, and improve the therapeutic effect. These findings demonstrated that by reducing the negative effects of 5-FU, such as weight loss and polyp burden, regulating intestinal inflammation, enhancing the intestinal epithelial barrier, and controlling the intestinal bacterial flora, pachymaran could significantly improve the anticancer effect of 5-FU. The therapeutic efficacy of this approach is also noteworthy (82).

In nanotechnology, Yang et al. found that graphene oxide nanosheets loaded with the model antigens ovalbumin and pachymaran and integrated nanocomplexes were efficiently phagocytosed by dendritic cells (DCs) through receptor-mediated endocytosis, which induced the maturation of DCs and improved the efficiency of delivery *in vitro* and *in vivo*. NPs also significantly enhanced OVA antigen-specific Th1 and Th2 immune responses *in vivo*, which significantly inhibited tumor growth in prophylactic therapy and achieved therapeutic efficacy in inhibiting established tumor growth, providing a promising strategy for enhancing antitumor immunity in cancer immunotherapy (83).

### 2.7 Ganoderma lucidum triterpenoids and polysaccharides

The two primary active ingredients that are isolated from the plant Ganoderma lucidum and used in traditional Chinese medicine are Ganoderma lucidum polysaccharide (GLP) and Ganoderma lucidum triterpenoids (GLT). It is the key component of the pharmacological effects of Ganoderma lucidum, with immunomodulatory, antitumor, anti-inflammatory, anti-aging, antioxidant, blood glucose regulatory, intestinal flora regulatory, and other pharmacological effects, and has important medicinal value in the treatment of cardiovascular and cerebral vascular diseases, central nervous system disease, and respiratory diseases (84–86). In recent years, the antitumor activity of GLPs and GLTs has become a hotspot of research by scholars at home and abroad, and they have obvious inhibitory effects on hepatocellular carcinoma and lung cancer.

Enhancing the resistance of tumor cells to drugs is a crucial factor in enhancing the efficacy of cancer therapy. Li et al. reported that GLPs could reverse multidrug resistance in the leukemia cell line K562/ADM and could significantly reverse the resistance of K562/ADM to DOX by reversing MDR by downregulating the expression of MDR-1 and MRP1 in K562/ADM cells (87). Yu et al. verified the sensitizing therapeutic effect of GLPs on hepatocellular carcinoma cells exposed to radiation. The results showed that GLPs enhanced radiation-induced growth inhibition and apoptotic death in HCC cells and inhibited the activity of DNA repair-related proteins in hepatocellular carcinoma cells under radiation conditions. By altering the Akt signaling pathway, GLPs improved the radiosensitization of hepatocellular carcinoma cells, indicating a possible therapeutic use for GLPs as radiosensitizers in the treatment of HCC (88). GLPs can effectively reduce the toxicity of chemotherapeutic drugs and reduce adverse effects on patients due to the toxic side effects of chemotherapeutic drugs. Qiu et al. used the coadministration of GLP and cisplatin for the treatment of lung cancer and found that GLP could effectively inhibit tumor growth and the formation of metastatic nodules in the lung tissues of mice, increase the survival rate of mice with lung cancer, and reduce the cytotoxicity of cisplatin in macrophages and normal lung fibroblasts (89).

Drug side effects may have a multifaceted impact on patients receiving tumor therapy and may cause psychosomatic damage and systemic symptoms in addition to directly affecting physiological health. Abulizi et al. reported that GLT attenuated cognitive dysfunction induced by the chemotherapeutic drug 5-FU blocked decreases in spatial and nonspatial memory, alleviated damage to hippocampal neurons and mitochondrial outcomes, increased 5-FU-induced hippocampal neuron survival, and increased the expression of mitochondrial biogenesis-related markers in the hippocampus, PGC-1α, and aberrant mitochondrial dynamics-related markers (MFN2, DRP1, and FIS1). GLT can prevent cognitive dysfunction in 5-FU-treated mice by preventing mitochondrial damage and promoting neuronal survival and growth, providing evidence for clinical studies of GLT as a promising adjunctive treatment for chemotherapy-related cognitive impairment (90). Chemotherapy-associated fatigue is a chronic state of weakness and exhaustion that occurs during or following chemotherapy treatment. A number of factors, including immune system suppression and the harmful side effects of the medications themselves, can cause this phenomenon. Chemotherapy-related fatigue significantly affects patients’ quality of life and treatment compliance, and effective management of chemotherapy-related fatigue is essential for improving patients’ quality of life and treatment outcomes. Abulizi et al. investigated whether GLT has anti-chemotherapy-related fatigue activity. Combined with the 5-FU treatment of CT26 hormonal mice to assess peripheral muscle and central fatigue-related behaviors, energy metabolism, and inflammatory factors, we found that GLT, by improving muscle mass and mitochondrial function, increased skeletal muscle glycogen content and ATP production and decreased lactate content and LDH activity. The inhibition of skeletal muscle p-AMPK, IL-6, and TNF-α expression ameliorated 5-FU-induced fatigue-like behavior in peripheral muscles. Moreover, GLT delayed 5-FU-induced central fatigue-like behavior by inhibiting the TLR4/Myd88/NF-κB pathway and downregulating the expression of hippocampal IL-6, iNOS, and COX2. The results showed that GLT could alleviate 5-FU-induced peripheral and central fatigue in tumor-bearing mice, which provided a rationale for the use of GLT as a potential therapeutic drug for chemotherapy-associated fatigue (91).

Chemotherapy-associated fatigue is a persistent weakness and tiredness that develops during or after chemotherapy. Numerous factors, such as immune system suppression and the negative side effects of the drugs, might cause this phenomenon. Zheng et al. designed a novel pH-sensitive nanoparticle drug delivery system based on GLP, which can deliver methotrexate and 10-hydroxy-camptothecin to tumor cells, thus exerting synergistic antitumor effects, and this nanoparticle showed better tumor inhibition and fewer side effects *in vivo*, making it a very promising antitumor treatment strategy (92).

### 2.8 Coix seed oil

Semen coicis is the dried mature seed kernel of Coix lacrymal-jobi L. var. ma-yuen (Roman.) Stapf of the grass family. It is a common Chinese medicine used for both food and medicine and has high nutritional value. Recent research has shown that Semen coicis has significant therapeutic effects on cancer, hypertension, hyperlipidemia, fatty liver disease, rheumatoid arthritis, and many other diseases. Modern pharmacological studies have shown that the main pharmacological substances that play an antitumor role exist in Coix seed oil (CSO). In addition, Coix seed polysaccharide polyphenols also have auxiliary therapeutic effects; CSO is used as a raw material to develop a new anticancer drug, Kanglaite injection, which is often used in clinical practice in combination with other anticancer drugs for the treatment of cancer (93).

CSO has a wide range of anticancer effects and has significant inhibitory effects on a variety of tumor cells. Abnormalities or mutations in PTEN, regarded as a tumor suppressor gene, are linked to the emergence and progression of numerous malignancies and play a role in controlling apoptosis. Yang et al. showed that in PANC-1 cells, the PTEN protein was involved in the process of CSO-induced apoptosis and mitochondrial damage. CSO regulates mitochondrial function by downregulating the expression of p-AKT, p-PI3K, and Bcl-2; upregulating the protein expression of the PTEN gene, caspase-9, Bax, caspase-3 and cytochrome c; and increasing the production of ROS, which induces apoptosis and inhibits tumor development in tumor cells (94). Studies have shown that CSO can inhibit the activity of HT-29 cells in a time/dose-dependent manner *in vitro* assays; CSO can induce apoptosis by modulating the PI3K/AKT signaling pathway, activating caspase-3, upregulating Bax and downregulating Bcl-2 (95). Currently, TNBC treatment is a clinical therapeutic challenge, and TNBC patients with high S1PR1 expression exhibit high proliferative activity and lymph node metastasis (96). Some scholars have applied CSO to explore the molecular mechanism of TNBC. Fang et al. found that *in vivo* experiments, miR-205–5p was significantly altered in the tumor tissues of mice after CSO intervention and sphingomyelin metabolism in the serum was elevated. miRNA-205 can directly target S1PR1 to regulate SM metabolism and cell proliferation. The cellular suppression of TNBC was achieved by downregulating the phosphorylation of STAT3, MAPK, and AKT, upregulating p27, and decreasing the expression of S1PR1 and cyclin D1, which was achieved through CSO intervention of miRNA-205 (97).

Screening for inhibitors of ABC transporter proteins is highly important for reversing MDR in tumors and improving the efficacy of tumor chemotherapy. Qian et al. tracked and examined the role of ABC transporter-mediated intracellular drug efflux. The results showed that in pancreatic cancer BxPC3 cells, Coix lacrymal extract intervention significantly increased the area under the curve (AUC), decreased the elimination rate constant (K), and increased the bioavailability of GEM while decreasing the expression of ABCB1 and ABCG2 drug-resistant proteins. The mechanism of synergistic enhancement of the therapeutic effect of GEM on pancreatic cancer cells after intervention with Coix lacrymal extract may involve altering the function of ABC transporter-mediated drug efflux (98).

Cancer cachexia is a common complication of tumors and greatly affects the quality and duration of patient survival. Liu et al. studied and explored cachexia and modeled cachexia in mice. On the one hand, in Lewis lung cancer cells, CSO intervention significantly alleviated weight loss and improved systemic inflammation and the weight and histological morphology of the gastrocnemius muscle and epididymal adipose tissue in mice; on the other hand, CSO reduced the expression of MuRF1 and the ratio of Ser536 to p65 in muscle tissue. In addition, it prevents cachexia and impedes the growth of malignant tumors by blocking HSL and AMPK activation induced by tumor cells (99).

In terms of nanoscale research, Transferrin-functionalized microemulsions containing CSO and tripterine coloaded with transferrin (Tf-CT-MEs) slowed tumors *in vivo* in a mouse model of HeLa graft tumors, and Tf-CT-MEs inhibited tumor cell proliferation, enhanced angiogenesis and induced apoptosis by regulating Bax/bcl-2 and caspase-3. Moreover, Tf-CT-MEs also reduced the serum concentrations of inflammatory factors (TGF-β1, CCL2, TNF-α, and IL-6), and Tf-CT-MEs enhanced tumor targeting, promoted the deep penetration of drug components, and improved the treatment of cervical cancer (100).

Some common broad-spectrum chemotherapeutic agents (e.g., PTX) still fail to achieve satisfactory therapeutic effects in clinical treatment due to their inherent defects (poor water solubility). It was discovered that a PTX-coix seed oil coloaded microemulsion (CP-ME) was created by combining CSO and PTX using nanotechnology. The results showed that in HeLa cells, the ratio of the percentage of apoptotic cells in the PTX intervention group to that in the CP-ME intervention group was 1:1.7; in a 3D tumor sphere model, CP-MEs penetrated more strongly in the HeLa 3D tumor sphere model, indicating that the water solubility of PTX was improved and that the antitumor effect was enhanced by the intervention of the CSO-containing nanodelivery system, and CP-MEs are expected to become a new method for the clinical treatment of tumors (101). Qu et al. further optimized coix seed component-based microemulsions (C-MEs) in their study to develop an octanoyl galactose ester-modified microemulsion system self-assembled from coix seed components (Gal(oct)-C-MEs) and applied this system to HepG2 hepatocellular carcinoma cells. The results of the cellular uptake assay showed that Gal(oct)-C-MEs had 2.28-fold greater cellular uptake than C-ME. The IC50 of Gal(oct)-C-MEs in HepG2 cells was 46.5 ± 2.4 μg/ml, which was significantly greater than that of C-MEs (132.5 ± 7.3 μg/ml). The intratumor fluorescence results of the mouse imaging experiments showed that the intratumor fluorescence after treatment with Cy5/Gal(oct)-C-MEs was 1.9-fold greater than that after treatment with Cy5/C-MEs. Stronger permeability and retention effects, as well as a greater ability to inhibit transplanted tumors in HepG2 nude mice, were demonstrated by the optimized Gal(oct)-C-MEs, suggesting promising potential for use in the treatment of liver cancer (102).

## 3 Chinese patent medicine

### 3.1 Kanglaite injection

Kanglaite injection (KLT) is an emulsion for intravenous and arterial infusion that is made using sophisticated preparation techniques from the traditional Chinese herb semen coicis. It is a new national class II anticancer drug. It is commonly used in the adjuvant treatment of lung cancer, liver cancer, and pancreatic cancer and the palliative treatment of advanced malignant tumors. Modern pharmacological research has shown that KLT not only inhibits cancer cells and efficiently relieves pain but also significantly improves immunity and can be combined with radiotherapy and chemotherapy to improve efficacy, reduce toxic side effects, and improve multidrug resistance in patients. KLT is an ideal drug for the comprehensive treatment of cancer in the clinic.

As a biphasic, broad-spectrum anticancer medication, KLTs exhibit synergistic benefits when used with chemotherapy and radiation therapy. Chemokinelike factor 1 (CKLF1) has chemotactic, colony-forming, and cell proliferation-promoting effects. In HepG2 cells, Cis-dichlorodiammine-platinum (CDDP) intervention significantly enhanced intracellular CKLF1 expression, and CKLF1 overexpression reversed the killing effect of CDDP on HepG2 cells, resulting in increased tumor cell viability and enhanced NF-κB activation, which aggravated the inflammatory response and promoted tumor progression, whereas KLT inhibited the expression of CKLF1 by reducing this pathway; on the other hand, after KLT combined with CDDP, the ABC drug efflux transporter proteins MDR1, MRP2 and BCRP were reduced in HepG2 cells, which promoted the sensitizing effect of CDDP and reversed CDDP-mediated chemoresistance to a certain extent (103). In animal trials, Cao et al. created a model of lung cancer bone metastases for group medication administration. The results showed that the rate of body mass improvement in the PTX monotherapy group was lower than that in the PTX+KLT combination group (*P*<0.05). After 21 d of treatment, the tumor area and tumor weight of the combination group were significantly lower than those of the single-drug group, suggesting that the combination of PTX and KLT is more effective than PTX alone in treating lung cancer bone metastases (104).

Herbal-derived KLT is considered an adjuvant therapeutic option for cancer treatment. A meta-analysis of EGFR-TKI/EGFR-TKI+KLT for the treatment of patients with stage III/IV NSCLC showed that compared with EGFR-TKI alone, KLT plus EGFR-TKI significantly improved the disease control rate (DCR), objective response rate (ORR), Karnofsky performance status (KPS), enhanced patient immunity, increased percentage of CD4^+^ T cells, CD4^+^/CD8^+^ ratio, and percentage of NK cells. The combination dramatically decreased the frequency of medication toxicity (vomiting and nausea) caused by EGFR-TKIs (105). The results of a meta-analysis showed that KLT in combination with gefitinib was superior to gefitinib alone in terms of increased objective remission rate, improved physical status, elevated percentage of CD4^+^ cells, natural killer cells, and CD4^+^/CD8^+^ ratio (106). A randomized controlled trial using KLT in combination with platinum-based chemotherapeutic (PBC) agents in patients with NSCLC (stage III/IV) showed that KLT plus PBC improved the DCR, ORR, 1-year overall survival (OS), QOL+t-cells, and CD4^+^/CD8^+^ ratio compared to PBC alone and that the combination therapy improved cellular immunity and attenuated severe toxicity caused by chemotherapy (59%) (107). In a randomized controlled clinical trial on patients with advanced pancreatic cancer (PC), compared with radiotherapy alone, KLT combined with radiotherapy significantly improved OS, overall response, and DCR and improved quality of life (QIR), the pain relief rate (PRR), and the weight gain rate (WGR). Radiation-related side events, including gastrointestinal symptoms, nephrotoxicity, leukopenia, thrombocytopenia, and myelosuppression, were less common in PC patients receiving KLT treatment (108).

A phase II trial with 46 patients examined the effectiveness of KLT in treating mucositis resulting from radiation therapy for head and neck malignancies. The results showed that the incidences of grade 3 mucositis, pain, dysphagia, and neutropenia were 10.9%, 2.2%, 10.9%, and 6.5%, respectively, while the incidence of grade 4 acute toxicity was zero. Opioid utilization was 2.2%. The radiation therapy dose was reduced by 2.2%, and there was no modification of the irradiation field. Cancer patients treated with KLTs have a low incidence of grade 3–4 radiation mucositis, no serious acute toxicity events, and good nutritional status and quality of life (109).

### 3.2 Xihuang Pill

Xihuang Pill (XHP) is an ideal anticancer CPM for clinical application. Current pharmacological research has demonstrated that XHP inhibits several cancer types, with a greater effect observed in solid tumors (110). It also plays an antitumor role in improving body immunity as well as regulating the tumor immune microenvironment (111).

Regulatory T cells (Tregs) are essential for the regulation of immune homeostasis. A large number of Treg cells exist in the tumor microenvironment and are currently considered to be the “main culprit” that helps tumor cells escape immune surveillance (112). Su et al. constructed a mouse 4T1 breast cancer model in their study, and the results showed that the total number of Treg cells in the tumor microenvironment decreased and the number of apoptotic Treg cells increases, and XHP regulates the expression of the Treg-related genes MEKK1, SEK1, JNK1, and AP-1, which inhibit breast cancer progression (113). Treatment of hepatocellular carcinoma cells with XHP reduced tumor cell viability and migration in a time- and dose-dependent manner. XHP activates proapoptotic proteins (Caspases 9 and 3) by regulating the PI3K/Akt/mTOR signaling pathway, promoting apoptosis and inhibiting the malignant progression of hepatocellular carcinoma cells (114). cAMP-PKA, an internal PKA in the classical pathway, is the primary cAMP effector molecule and is crucial for controlling bioactive responses and cellular homeostasis. Several studies have demonstrated a significant association between XHP and the cAMP/PKA signaling pathway. In breast cancer cells, the proliferation, migration, and invasion of breast cancer cells were significantly inhibited, and apoptosis was induced after XHP intervention. XHP inhibits the malignant progression of breast cancer cells by activating related genes by regulating the cAMP/PKA signaling pathway (115). XHP significantly inhibited the formation of mimetic ducts in gastric cancer (MCC-803) cells. A reduced number of mimetic ducts formed in mouse gastric cancer tissues. XHP decreased the malignant progression of gastric cancer and prevented the generation of angiogenic mimics in this disease (116).

The most common malignant tumors in the central nervous system are called gliomas. Studies have shown that in primary glioma models (U251 and SHG-44), XHP treatment alone downregulated POU4F1 and STAT3 levels and increased LDH release and IL-1β and IL-18 levels, and the apoptosis-associated factor NLRP3 was activated. XHP promotes cellular pyroptosis by regulating the POU4F1/STAT3/NLRP3 pathway and inhibits glioma malignant progression (117). A substantial amount of data points to the involvement of glioblastoma stem cell-like cells in the development of this disease and its unfavorable prognosis in glioblastoma multiforme (GBM). In addition to causing apoptosis, XHP reduced the number of GBM 3D spheroids, the proportion of CD133-positive cells, and the expression levels of tumor stem cell markers (CD133 and SOX2) *in vitro* in the U87/U118 cell model. XHP downregulated tumor stem cell markers through modulation of the CD133/EGFR/Akt/mTOR pathway and inhibited tumor stem cell enrichment and self-renewal, preventing the malignant progression of glioma (118). In studies combining XHP with other antitumor agents, we found that in glioblastoma (U87 and U251), Ki67 and PCNA expression was significantly suppressed, the Bcl-2/Bax ratio was downregulated, and there was no significant *in vivo* toxicity of the drug in the XHP combined with motezolomide group compared with the XHP or motezolomide monoadministration group, suggesting that XHP enhances the proapoptotic effect of motezolomide by inhibiting the Akt/mTOR pathway (119).

Anlotinib is a novel small-molecule multitarget tyrosine kinase inhibitor that exerts antitumor angiogenesis and tumor growth inhibition effects. Using a Lewis lung cancer (LLC) mouse model, Cao et al. investigated the transcriptomic and gut microbiota regulatory effects of XHP in combination with anlotinib. The results showed that XHP increased the proportion of the beneficial bacteria Bacteroidetes and Pseudomonas and that XHP played a key role in regulating the gut microbiota. The transcriptomics results suggested that differential gene expression after XHP intervention was associated with biological processes involved in angiogenesis (regulation of blood vessel diameter). When combined, XHP and anlotinib have an increased anticancer impact by modifying the composition of the gut microbiota and tumor angiogenic pathways, suggesting that XHP could be a further treatment option for LLC (120).

### 3.3 Compound Banmao capsule

Compound Banmao capsules (CBMC), a commonly used clinical antitumor drug in Chinese hospitals, include Cantharides, ginseng, Astragalus membranaceus, and 11 other Chinese herbs, of which the main drug is Cantharides, which is commonly used to treat primary liver cancer, lung cancer, colorectal cancer, and stomach cancer. Studies have shown that CBMC, as a proprietary Chinese medicine, has the advantages of enhancing immunity and alleviating antitumor side effects, and its advantages are even more obvious when applied in combination with other antitumor drugs.

Sun et al. investigated the effect of CBMC intervention on the survival and apoptosis of gastric cancer cell lines. They showed that in BGC-823 and SGC-7901 cells, CBMC combined with 5-FU had an inhibitory effect on both cell lines and was able to significantly reduce the level of c-Myc mRNA expression, increase the level of p53 mRNA expression and promote apoptosis through the regulation of the p38/JNK protein pathway.

The combined medication had a far stronger effect than either medication alone. CBMC could enhance the killing ability and viability inhibition of gastric cancer cells by 5-FU and inhibit the malignant progression of gastric cancer (121). Fluorouracil, platinum, PTX, and oncocyclic agents are the mainstays used for the treatment of advanced gastric cancer, but there is no standardized protocol for first-line chemotherapy for advanced gastric cancer because of the heterogeneity of gastric cancer. Presently, advanced gastric cancer patients are treated with PTX plus 5-FU and calcium folinic acid chemotherapy (TFL regimen), which has proven to be more effective. Chemotherapy can lead to immunocompromise in patients, which is mainly dominated by the dysregulation of the number or ratio of T lymphocyte subpopulations, and improving the immune function of tumor cancer patients is of great clinical significance for improving the tolerance of chemotherapy and improving the prognosis. A clinical study showed that TFL+CBMC treatment was superior to a single TFL regimen in terms of clinical efficacy, pre- and posttreatment serum T-lymphocyte subpopulation levels, and tumor marker (CA199, CEA, CA724) expression levels. The results showed that the disease control rate was greater in the combination group (53.85% *vs*. 30.77%). Serum CD4^+^/CD8^+^, CD3^+^, and CD4^+^ levels were greater, and CA199, CEA, and CA724 levels were lower in the combination group than in the conventional group. To cope with advanced gastric cancer, CBMC combined with a TFL regimen is superior to a single TFL regimen, and CBMC, as a complementary treatment to chemotherapy, has potential value and therapeutic advantages in enhancing body immunity and lowering tumor markers (122).

The primary treatment for patients with advanced colon cancer is chemotherapy. The chemotherapy regimen consisting of oxaliplatin, 5-FU, and calcium levofolinate (mFOLFOX6) has been widely used with remarkable efficacy and has gradually become a globally recognized standard regimen for the treatment of colon cancer. More focus should be placed on enhancing the quality of life and reducing pain for patients with advanced malignancies to increase their chances of survival. A clinical study on advanced colorectal cancer showed that the overall effective rate of treatment was greater in the CBMC^+^ mFOLFOX6 combination group (77.78% *vs*. 57.50%). The Karnofsky Performance Scale (KPS) score is a score that reflects the functional status and activity of tumor patients, and a higher score indicates a greater tolerance to chemotherapy and a better state of health. The Karnofsky Performance Scale (KPS) scores of the two groups were greater after treatment than before treatment, and the increase in the combination group was greater than that in the single chemotherapy group. The combination group had higher levels of CD3^+^, CD4^+^, and CD4^+^/CD8^+ cells^ and a lower incidence of side effects such as platelet drop, leukocyte drop, and gastrointestinal reactions. According to the results mentioned above, CBMC-assisted mFOLFOX6 treatment can significantly enhance autoimmunity and chemotherapy tolerance and improve quality of life and clinical symptoms in patients with advanced colon cancer (123). Chen et al. explored the efficacy and effects of CEA, VEGF, and CA199 in colorectal cancer patients using CBMC combined with Gimeracil and Oteracil Porassium Capsules (S-1) in their study. The results showed that the ORR and DCR were greater in the combination group than in the single chemotherapy group (53.33% *vs*. 43.33% and 70.00% *vs*. 33.67%, respectively). The CEA, VEGF, and CA199 levels decreased more significantly in the combination group than in the single chemotherapy group. The CD3^+^, CD4^+^, NK, and CD4^+^/CD8^+^ levels were significantly reduced in the single chemotherapy group, while they were upregulated in the combination group. Therefore, the use of CBMC combined with S-1 monotherapy in the treatment of advanced colorectal cancer has better efficacy, can significantly improve the quality of life of patients and reduce the levels of CEA, VEGF, and CA199, and is worthy of clinical popularization and application (124).

The preferred treatment for middle- and late-stage primary hepatocellular carcinoma is transcatheter hepatic artery chemoembolization (TACE). However, its use is limited by the damage to liver function caused by multiple interventions. The combination of traditional Chinese herbs can effectively improve liver function following surgery (125). Therefore, it is urgent to explore herbal adjuvant therapies that can alleviate TACE-induced liver injury. Cantharidin is the main active ingredient of CBMC, and relevant studies have shown that cantharidin can inhibit DNA and RNA synthesis, has a strong affinity for liver and cancer cells, is effective against primary liver cancer and other cancers, and has no myelosuppressive effect (126). Zhao et al. explored the efficacy of CBMC combined with TACE in the clinical treatment of primary liver cancer. The results showed that the solid tumor control rate was better in the combination group (92% *vs*. 75%), the immunity-related indices were better in the combination group (CD3^+^, CD4^+^, CD8^+^), and the AFP in the combination group was significantly lower than that in the single TACE group. The *in vitro* results showed that CBMC could play an anticancer role by inhibiting the expression of the C-Mc gene and promoting the expression of the p53 gene. In conclusion, the combination of CBMC and TACE can greatly increase the therapeutic impact, strengthen the patient’s immune system, and stop the growth of hepatocellular carcinoma cells (127).

The formation of portal vein thrombi in hepatocellular carcinoma is an important factor affecting the prognosis of patients with this disease. Liu et al. explored the impact of applying CBMC to treat the 6-month survival of patients with advanced hepatocellular carcinoma with VP3–4 portal vein tumor thrombus (PVTT). The results of the study showed that at 6 months, the overall survival rate and median survival time of patients in the CBMC group were greater than those in the monotherapy group (4 *vs.* 2.2 months). When paired with PVTT, the use of CBMC can increase patient benefits and increase the survival duration of patients with hepatocellular cancer (128).

Cervical cancer is a common clinical tumor of the reproductive system. Chemotherapy is a key tool for preventing cervical cancer metastasis and recurrence after surgery, but the main cause of patients stopping treatment midway through chemotherapy is the numerous side effects of the drug. Li et al. explored the application value of CBMC in postoperative radiotherapy for cervical cancer. The results showed that the total effective rate of CBMC in the combined group was greater than that in the single radiotherapy group (87.50% *vs*. 70.3%), the levels of tumor markers (CA125 and CEA) were significantly lower, and the levels of immune-related indices (CD3^+^, CD4^+^, and CD4^+^/CD8^+^) were significantly greater in the combined group. Postoperative radiotherapy combined with CBMC adjuvant therapy for cervical cancer can improve patient immunity, reduce tumor marker levels, improve antitumor efficacy, and inhibit the malignant progression of tumors (129).

In other tumors, CBMC also has significant antitumor advantages. In NSCLC, CBMC can increase the CD4^+^ and CD8^+^ levels in the peripheral blood of patients with intermediate and advanced NSCLC to improve cellular immunity (130). Patients’ quality of life and immune system performance were greatly enhanced when CBMC and gefitinib were used together, increasing the treatment efficacy from 53% to 72% (131). In osteosarcoma cells, CBMC combined with apatinib significantly inhibited tumor growth in an OS nude mouse model, attenuated weight loss due to tumor growth, and reduced VEGF and Bc1–2 expression in a dose-dependent manner. By blocking the VEGF, Bc1–2, and PI3K/AKT pathways, CBMC, in combination with apatinib, prevented the malignant progression of osteosarcoma (132).

### 3.4 Kanglixin capsule

Kanglixin capsules (KLXs) are a new class of anti-malignant tumor preparations that are pure plant-based and are used to treat digestive tract tumors, lung cancer, breast cancer, and other cancers. Using Tibetan medicine and modern scientific research, the anticancer-active ingredients of the capsules were extracted.

Chemotherapy, in which fluorouracil+platinum cytotoxic drugs are used as the basic drugs, is one of the main treatments for intermediate and advanced gastric cancer, and the SOX regimen (oxaliplatin+S-1) is the first-line regimen for intermediate and advanced gastric cancer treatment. Due to the significant side effects, combination therapy, including numerous anticancer chemotherapeutic agents, is debatable. It is also critical to investigate adjuvant medications to mitigate the harmful side effects of chemotherapeutic agents. A clinical study using KLX in combination with SOX for the treatment of intermediate and advanced gastric cancer showed that the overall effective rate in the KLT+SOX combination group was greater than that in the single SOX group (86.67% *vs*. 63.33%). The levels of tumor markers (CA19–9, CA12–5, and CA72–4) decreased more in the combination group, the proportions of T lymphocytes and NK cells in the combination group were greater than those in the single SOX group, and the combination group had a lower incidence of adverse reactions (16.67% *vs*. 26.67%). Compared with the SOX regimen alone, the combined application of KLX for the treatment of middle and advanced gastric cancer can improve treatment efficacy and has a positive effect on improving the immune function of patients, reducing adverse reactions and other aspects (133). Li et al. reported that the FOLFOX4 regimen combined with KLX adjuvant therapy had strong antitumor effects on tumor markers, body immunity, and Karnofsky Performance Scale (KPS) scores and outperformed chemotherapy alone in the treatment of intermediate and advanced gastric cancer (134). CIK cells are cytotoxic T cells that strongly kill T cells and NK cells. CIK cells have a good killing effect on drug-resistant tumor cells. DC cells can express a variety of antigenic peptides and secrete a large number of IL-2 and IFN-γ anticancer factors, which promote the proliferation and maturation of CIK cells and enhance their anticancer activity (135). Wang et al. reported that the immune-related indices (CD4^+^/CD8^+^) and incidence of adverse reactions in the combination group were greater than those in the single treatment group and that KLX combined with DC-CIK could significantly improve the immune function of patients with advanced gastric cancer, alleviate clinical symptoms, mitigate the toxic side effects of chemotherapeutic drugs, and delay tumor progression (136).

KLX can be used as an adjuvant and complementary anticancer medication to treat other malignancies by acting as an antitumor agent in combination with chemotherapeutic agents. In NSCLC, the pemetrexed+cisplatin regimen (137) and PTX+cisplatin regimen (138) are commonly used chemotherapeutic regimens with good clinical efficacy, and after combination with KLX, they can effectively prolong the survival time of patients, improve the quality of life of patients, have fewer adverse reactions, have high safety, and significantly enhance the antitumor therapeutic effect. Her-2-negative advanced breast cancer patients treated with anthracyclines and traditional PTX drugs can develop treatment resistance, visceral crisis, and rapid disease progression, leading to treatment failure (139). Wang et al. explored the clinical efficacy of KLX combined with albumin (PTX) in Her-2-negative advanced breast cancer patients. The results showed that the objective remission and disease control rates of patients in the combination group were significantly greater than those in the single chemotherapy group (62.50% *vs*. 41.67%, 89.58% *vs*. 72.92%), the median progression-free survival of patients in the combination group was greater (5.23 months *vs*. 2.85 months), and the incidence of adverse events in patients in the combination group was significantly lower than that in the single chemotherapy group (33.33% *vs*. 97.92%). According to previous research, KLX can successfully increase the progression-free survival of patients with advanced Her-2-negative breast cancer, decrease the serum levels of the tumor markers CEA and CA153, decrease the VAS score, and prevent malignant progression. It can also lessen the likelihood of chemotherapy-related side effects, increase patient survival rates, and has good safety and feasibility (140).

One of the most widely used therapies for esophageal cancer in clinical practice is synchronous irradiation, which has numerous drawbacks, including toxic side effects, despite its remarkable efficacy. Zuo et al. showed that the total treatment efficiency in the KLX combined with the radiotherapy group was significantly greater than that in the conventional radiotherapy group (43.3% *vs*. 16.7%). However, there was no statistically significant difference in the occurrence of radiotherapy complications or radiotherapy side effects between the KLX group and the conventional radiotherapy group (13.3% *vs*. 16.7% and 43.3% *vs*. 46.7%, respectively). Patients with esophageal cancer can benefit from the application of KLX for radiation sensitization treatment, which can effectively increase the therapeutic efficacy of radiation therapy and improve patient outcomes (141).

### 3.5 Xiaoaiping Injection

Xiaoaiping Injection (XAP) is made from the dried vine stem of the traditional Chinese herb Marsdenia tenacissima by a modern process of water extraction and alcohol precipitation. Modern pharmacological research shows that XAP has obvious antitumor activity and is often used alone or in combination with chemotherapy and radiotherapy for the treatment of gastric cancer, lung cancer, and other tumors, with remarkable efficacy and high safety.

Abnormalities in the expression of miRNAs, including miR-224 and miR-195, have a significant impact on the growth, invasion, metastasis, and other pathological processes of tumor cells (142–144). CT dynamic enhancement scanning can provide technical evidence-based imaging support for evaluating the blood perfusion of tumor lesions and determining the degree of tumor malignancy. Deng et al. found (145) that XAP combined with gefitinib was superior to gefitinib alone in regulating serum miR-224 and miR-195 levels in patients with advanced lung cancer and that the combination treatment improved the blood perfusion of tumor foci in patients with advanced lung cancer, with an overall effective rate of 60.47% (37.21% in the single treatment group), and that the combination with gefitinib blocked tumor vascular proliferation and reduced the tumor cell blood supply. Fibrinolytic, anticoagulant, and coagulation dysfunction in lung cancer patients varies (146), and APTT, PT, D-D, and FIB are often employed as indicators for the clinical evaluation of coagulation function. In this study, we found that XAP combined with gefitinib improved the coagulation function of patients, corrected the imbalance of the coagulation system, and reduced the risk of thrombosis by regulating the above indicators. In summary, XAP combined with gefitinib can regulate the proliferation of many kinds of cells and directly inhibit the growth of cancer cells. It contributes to individuals with advanced lung cancer having a higher survival rate.

Ovarian cancer is a common gynecological tumor, and PTX is a commonly used and effective drug for the treatment of ovarian cancer. The pregnancy X receptor (PXR) is a member of the nuclear receptor superfamily. In addition to being crucial in controlling the body’s xenobiotic/endogenous chemical metabolism and excretion, its primary task is to detect the presence of external, harmful compounds. Pregnane X receptor activation can affect the efficacy of chemotherapeutic drugs and cause chemoresistance, so targeting the pregnane X receptor may be a new strategy to improve the pharmacokinetics of chemotherapeutic drugs. In ovarian cancer SK-OV-3 cells, XAP enhanced the antiproliferative and proapoptotic effects of PTX on tumor cells and inhibited PTX-induced PXR and the antiapoptotic protein Bcl-2, exerting antitumor effects and inhibiting the malignant progression of ovarian cancer. Patients with ovarian cancer respond better to treatment when XAP and PTX are combined (147).

The activating transcription factor 3 (ATF3) gene is involved in the cell cycle and immune regulation. The ATF3 gene is overexpressed in cancer cells, and ATF3 overexpression enhances tumor motility and invasiveness. ATF3 is overexpressed in breast cancer cells treated with PTX, and XAP reverses the effects of PTX on ATF3. *In vivo*, experiments also confirmed that the combination enhanced the inhibitory effect on tumor cells. These results suggest that XAP enhances PTX efficacy by inhibiting ATF3 (148). TNBC is a special subtype of breast cancer and is a refractory tumor. Epirubicin is a commonly used clinical chemotherapy regimen. Lin et al. applied epirubicin combined with XAP to explore the efficacy of TNBC treatment, and the study showed that after 12 weeks of treatment, the total clinical efficacy rate of the combination group was greater than that of the single chemotherapy group (91.11% (41/45) *vs*. 63.64% (28/44)). After 12 weeks of treatment, the combination group had better immune-related indices and lowered tumor marker levels. For TNBC patients, XAP, in conjunction with epirubicin neoadjuvant chemotherapy, has a demonstrable therapeutic impact, lowers serum tumor marker levels, and enhances the immune system and quality of life (149).

We treated BGC-823 and MGC-803 cells with either S-1 alone or S-1 plus XAP to examine the synergistic effect of XAP on S-1 in gastric cancer cells. Compared with those in the single-agent S-1 group, the combination treatment significantly inhibited cell proliferation, cell adhesion ability, and cell metastasis by downregulating the expression of VEGF, MMP-9, N-cadherin and vimentin and elevating the expression of E-cadherin, which affected EMT (150). The liver is a common metastatic organ of gastric cancer. When gastric cancer patients with liver metastasis are in the advanced stage of cancer and have a poor prognosis, chemotherapy is the main treatment modality. Like other chemotherapeutic regimens, the combination of oxaliplatin and capecitabine, or CapeOX, is a frequently used chemotherapy regimen. However, this modality has evident harmful side effects that seriously impair patients’ quality of life. The study showed that the disease effectiveness rate (59.57%) and DCR (93.62%) of the XAP+CapeOX combination group were greater than those of the chemotherapy-only group (38.10%, 78.57%), the expression of invasive genes in tumor tissues (MMP-2,7,9) was lower in the combination group, and adverse reactions, such as leukopenia, platelet drop, and hand-foot syndrome, were less common in the combination group than in the chemotherapy-only group. The above results indicate that XAP combined with CapeOX is an effective regimen for the clinical treatment of stage IV gastric cancer patients with liver metastasis (151). In China, the first-line recommended treatment for systemic chemotherapy for advanced gastric cancer is oxaliplatin plus the S-1 (SOX) regimen. Clinical observation of XAP combined with the SOX regimen in the treatment of advanced gastric cancer revealed that three patients with advanced gastric cancer without surgical indications at the initial diagnosis in the XAP+SOX group achieved improved survival through comprehensive antitumor treatment combined with the XAP+SOX regimen and the tumors receded significantly and were successfully transformed into surgically resectable tumors after resection of the primary or metastatic tumors. In advanced gastric cancer, XAP, in conjunction with the SOX regimen, may be one of the most effective strategies for achieving long-term disease-free survival, offering a valuable avenue for translational therapy investigation (152).

Patients with gastric cancer who develop ascites have an unconfined tumor, distant metastases, malignant progression, and an advanced stage of the illness, necessitating aggressive symptomatic therapy to prolong patient survival. Xu et al. used Wistar rats for experimental modeling, and the results showed that after XAP intervention, the number of ascites was reduced, the survival rate of tumor cells was reduced, immune-related proteins were increased (CD4^+^ and CD8^+^); the expression of inflammatory factors was reduced (IL-1β, IL-2, and IL-4); and TGF-beta1 and Smad2 were downregulated in rats. These findings indicate that XAP can significantly reduce the number of ascites, inhibit the proliferation of intraperitoneal tumor cells, and improve the immune status of rats and is an effective regimen for inhibiting the malignant progression of tumors (153). Thrombocytopenia after chemotherapy is a common chemotherapy-related side effect, and chemotherapeutic drugs can inhibit the hematopoietic function of the bone marrow, leading to thrombocytopenia. A randomized controlled clinical trial of XAP for the treatment of chemotherapy-induced thrombocytopenia. According to the findings, platelet counts in the XAP group began to rise after 21 days of involvement. For patients with postchemotherapy thrombocytopenia, XAP offers a safe and efficient treatment alternative as well as a clinical program (154).

### 3.6 Elemene Injection

Elemene Injection (EI) is extracted from Chinese herbs and injected intravenously into emulsions after special treatment. It has a potentiating impact on chemotherapy and radiation therapy and can effectively prevent the proliferation of cancer cells. The main component of EI is a mixture of β-, γ-, and δ-elements. As an antitumor phytochemical drug independently developed by our country, EI was approved by the state as a new class 2 antitumor drug in 1995 and was successfully listed on the market. A large number of experimental studies and clinical practice have further confirmed that EI has a broad antitumor spectrum, precise clinical efficacy, and few side effects and can cross the blood-brain barrier (155, 156). Currently, EI is frequently utilized to treat brain metastases and a variety of solid malignancies.

Breast cancer is currently one of the most common malignant tumors in women, and brain metastasis occurs in 10%~16% of patients during the treatment of breast cancer (157). Radiotherapy is an effective treatment for patients with brain metastasis. Yang et al. used EI combined with radiation therapy to treat brain metastases of breast cancer patients with some degree of efficacy. The results of the study showed that the combined treatment group (EI+whole brain radiotherapy) relieved clinical symptoms faster than the whole brain radiotherapy group alone (clinical symptom relief rate was 75% *vs*. 54.5%, respectively), the effective rate (CR+PR) was 56.3% *vs*. 21.4%, and the occurrence of leukopenia was lower than that of the combined group than that of the whole brain radiotherapy group alone. In the treatment of brain metastases from breast cancer, combining EI with whole-brain radiation has a synergistic impact that can increase patients’ near-term efficacy (158).

Prostate cancer (PCa) is the most common malignant tumor of the male genitourinary system, and bone is the most common metastatic site of PCa and the main cause of death; PCa bone metastasis often suggests a poor prognosis. Prostate-specific antigen (PSA) is a kind of antigen related to the prostate. In prostate cancer patients, the expression of PSA and f-PSA is increased, which is highly important for the diagnosis and treatment of prostate cancer. The PSA level is an independent prognostic factor (159). The extent of PCa-induced metastatic bone disease is indicated by the expression of alkaline phosphatase, an enzyme that is surface-expressed by osteoblasts and is frequently used to predict the prognosis and course of treatment for patients with PCa bone metastases (160). Zoledronic acid is the most commonly used drug in patients with PCa bone metastases. Qiao et al. investigated the clinical efficacy of EI combined with zoledronic acid in the treatment of PCa bone metastasis. The results showed that after 4 cycles of treatment, compared with those of the zoledronic acid group, the combination (EI+zoledronic acid) group had significantly lower VAS scores, the Karnofsky Performance Scale (KPS) scores of the combination group were greater than those of the zoledronic acid group, the total clinical efficacy rate of the combination group was greater (70.00% *vs*. 30.00%), and the water content of the serum indices of the combination group was lower than that of the zoledronic acid group. The results indicate that zoledronic acid and EI work synergistically and that the combined use of the two drugs is more clinically effective in treating PCa bone metastases (161).

A meta-analysis of hepatocellular carcinoma treated with EI and TACE. The findings demonstrated that the ORR and OS of the EI+TACE group were significantly better than those of the TACE group alone and that EI increased the therapeutic efficacy and survival rate of TACE. These findings warrant a more thorough investigation (162). Another meta-analysis revealed that EI combined with platinum-based chemotherapy (PBC) achieved significant results in the treatment of stage III/IV NSCLC. The EI+PBC regimen improved the DCR, ORR, 1- and 2-year survival, QOL, and immune-related indices (CD4^+^, CD4^+^/CD8^+^), indicating that EI can enhance the therapeutic effect of PBC and strengthen the body’s immunity, which is conducive to the continuation and smooth implementation of antitumor therapy and prolongs patient survival (163).

### 3.7 Weimaining capsule

Radiation, chemotherapy, and molecular targeted therapy are all beneficial for patients with advanced lung adenocarcinoma. Chinese herbs have also shown good clinical performance in treating this disease. Weimaining capsules (WMNs), which are made from the extracted and purified rhizome of the Chinese herb buckwheat, can improve the clinical efficacy of lung cancer treatment, reduce the adverse effects of radiotherapy and chemotherapy, and improve quality of life. WMNs may be used alone in patients with lung cancer who are not candidates for chemotherapy or radiation therapy.

The epidermal growth factor receptor (EGFR) driver gene is mutated in approximately one-third of lung cancer patients (164). EGFR tyrosine kinase inhibition (EGFR-TKI) has been included in the guidelines as a first-line treatment option for patients with EGFR driver-positive advanced lung cancer, and the clinical efficacy of ositinib, a 3rd-generation EGFR-TKI targeted drug that acts on T790M-positive mutations, has been confirmed in a wide range of clinical trials. However, after 9.6 months of oral ocitinib treatment, patients with advanced lung adenocarcinoma and EGFR T790M-positive mutations invariably exhibit acquired resistance to ocitinib, which results in disease progression and impacts the clinical prognosis of patients (165). Zhou et al. explored the efficacy of WMN augmented with ositinib in the treatment of lung adenocarcinoma. The results showed that after 1 month of treatment with WMN combined with ositinib, the overall efficacy rate was similar in the combination group (WMN+ositinib). In the OS group (20.00% *vs*. 16.67%), the DCR in the combination group was greater than that in the monotherapy group (73.33% *vs*. 46.67%). Disease progression-free survival was longer in the gefitinib group than in the osimertinib group [(11.40 ± 0.20) months *vs*. (10.67 ± 0.28) months]. Patients with advanced lung adenocarcinoma with an EGFR T790M mutation may benefit from longer progression-free survival and a greater rate of disease control when WMN and oxitinib are combined (166). The GP regimen (GEM+cisplatin) is the first-line regimen for chemotherapy in patients with advanced NSCLC, and its clinical benefits are widely recognized, while serious drug resistance exists, making it difficult for some patients to achieve considerable efficacy after chemotherapy and significantly decreasing their quality of life. Cellular immunity mediated by T lymphocytes and NK cells is an important mechanism by which the body fights against tumors. The inflammatory microenvironment also promotes the progression and metastasis of lung cancer, and TNF-α CRP and IL-6 are common inflammatory factors, and their serum levels are generally elevated in lung cancer patients and are significantly correlated with tumor activity. Several researchers have investigated the therapeutic effectiveness of WMN in conjunction with a GP regimen for treating NSCLC. The results showed that the objective tumor efficacy rate of 63.27% and clinical benefit rate of 89.79% in the combination group (WMN+GP) were greater than those of 32.65% and 65.31%, respectively, in the GP group. The immunity-related indicators of the combined group were greater than those of the GP group, and the incidence rates of grade II and III adverse reactions were 7.46% and 4.48%, respectively, which were lower than those of the GP group (25.37% and 14.92%, respectively). The serum levels of TNF-α, CRP and IL-6 in the combined group were lower than those in the GP group after treatment, indicating that WMN can, to a certain extent, restore the immunosuppression produced by the GP regimen, inhibit the undesirable progression of the inflammatory microenvironment, reduce complications, lower postoperative recurrence, and effectively improve the clinical effect of GP treatment for lung cancer patients, which has positive clinical significance (167, 168).

Breast cancer is regarded as a systemic disease that is highly susceptible to lymphatic and hematogenous metastases. Chemotherapy is a key component of this type of treatment, and the clinical selection of an appropriate chemotherapy regimen focuses on the trade-off between the drug’s effectiveness and toxicity. Capecitabine, an oral fluorouracil-based complex, is commonly used to treat advanced breast cancer. Wang et al. studied the efficacy and safety of WMN combined with capecitabine in the treatment of advanced breast cancer. The results showed that no patient experienced grade IV or higher adverse reactions, and no patient completely interrupted treatment because of adverse reactions, indicating that the safety and tolerability of WMN combined with capecitabine are better, which can help to protect the survival quality of patients. The WMN combined with the capecitabine program has a precise effect and mild adverse reactions, which are within the tolerable range of patients, and has a very positive significance in promoting the recovery of patients and improving their quality of life (169).

## 4 Discussion

**Figure 1:**
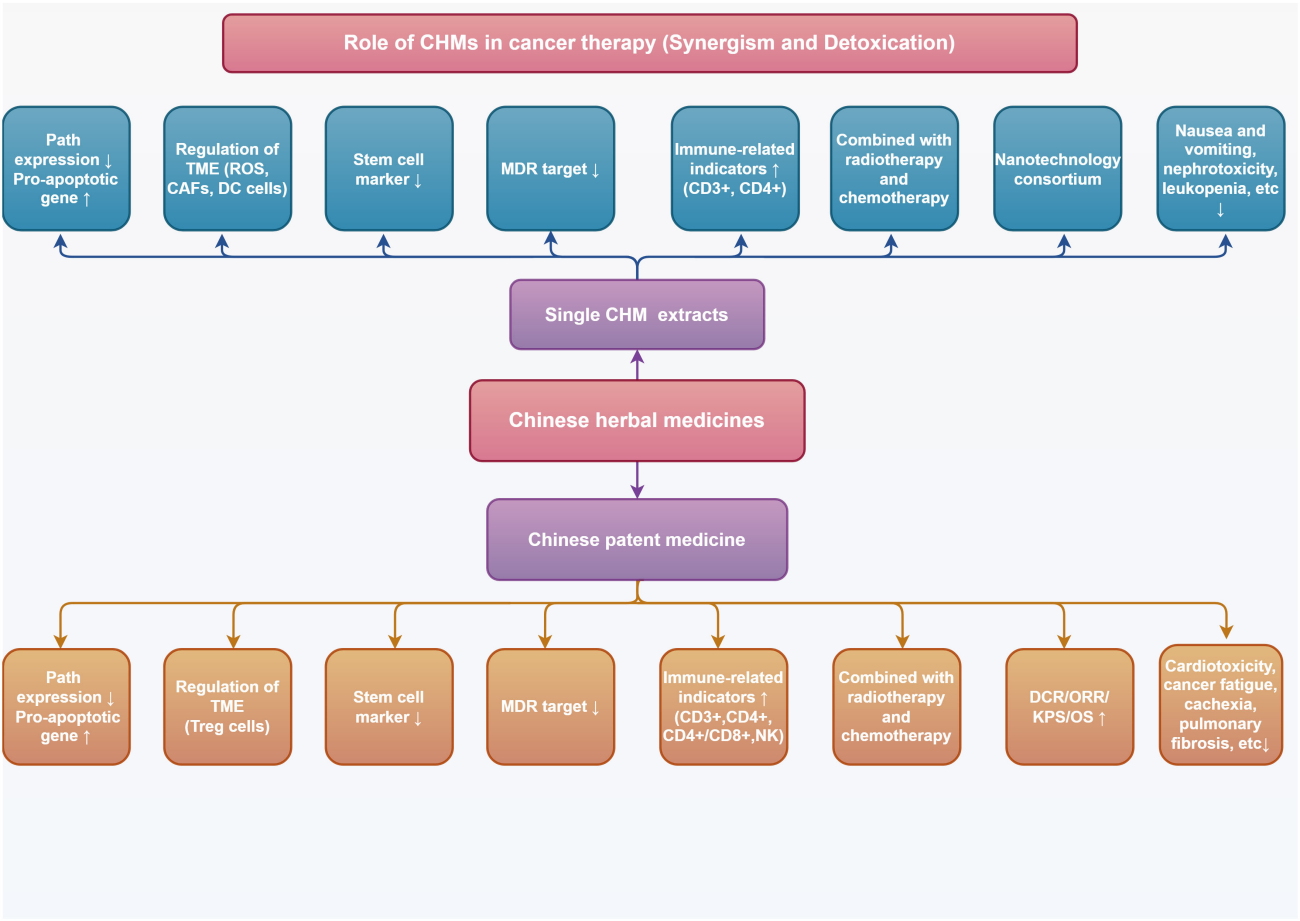
Role of CHMs in cancer therapy (Synergism and Detoxication) [Image credit: By Figdraw (https://www.figdraw.com)].

A malignant tumor is usually defined as a complex systemic chronic disease, and mainstream allopathic medicine, such as radiotherapy, chemotherapy, immunotherapy, and targeted therapy, is an effective treatment for middle- and late-stage malignant tumors. When conventional allopathic medicine is applied to treat cancer, patients experience unbearable, unpleasant responses and toxic side effects that have a significant negative impact on their anticancer treatment, sometimes resulting in treatment discontinuation. The use of CHM and CPM in antitumor therapy has received increasing research and clinical attention. As an important part of CAM, it has incomparable advantages and remarkable therapeutic effects in combination with mainstream allopathic medicine, improving its therapeutic effects and alleviating adverse reactions. This review summarizes recent research progress in the field of traditional Chinese medicine on the efficacy and toxicity reduction of CHM and CPM to complement mainstream allopathic medicine. This review focuses on basic research and clinical observations of the ability of TCM to alleviate adverse effects and enhance treatment efficacy. They inhibit the malignant progression of tumors, improve patients’ quality of life and prolong survival time by modulating multiple mechanisms to fight against tumors, including inducing apoptosis of tumor cells, inhibiting proliferation of tumor cells, blocking angiogenesis of tumors, enhancing immune responses, and synergizing with Western drugs to enhance efficacy and improve adverse events (**Figure 1**).

This paper reviews the antitumor-effective monomers of CHM and the drug composition of CPM, as well as their mechanisms of action, target cancer species and anticancer effects (**Table 1**). Chinese herbs contain a variety of anticancer active ingredients and have inhibitory effects on various cancer types; they can synergize with a variety of anticancer drugs to enhance drug sensitivity and alleviate drug-induced adverse reactions; and can be combined with a variety of novel nanodelivery systems to enhance the immune function of the organism and enhance antitumor effects.

**Table 1:** Representation and summary of the anti-tumor effects of Chinese medicines/proprietary Chinese medicines.

Name	Principal active ingredient/drug composition	Path/target	Type of cancer	Antitumor effect	*Ref.*
Ginseng	Ginsenosides, Rg1, Rg3, Rg5, Rb1, Rh2	p21, TP53, Bax, PFKFB3, mTOR/HIF-1α/VEGF, ZFP91, PI3K/Akt, Wnt/β-catenin, Hedgehog, NF-κB	Breast, osteosarcoma, pancreas, liver, stomach, hypopharynx, cervix, lung, kidney, and colorectal cancer	①Enhanced sensitivity to adriamycin, gemcitabine, sorafenib, cisplatin, etc. and sensitization to radiotherapy. ②Reduction of drug-induced side effects such as neuropathic pain, cardiotoxicity, etc. ③Combining with multiple nanodelivery systems to enhance anti-tumor effects	(7–35)
Largehead atractylodes rhizome	Atractylenolide I, II, III	Hsp27/eIF4E, XIST/miR-30a-5p/ROR1, STAT3/PKM2/SNAP23, Nrf2/NQO1/HO-1, CTGF	Prostate, breast, and colorectal cancer	①Enhancement of drug sensitivity of tumor cells to cabozantinib and paclitaxel. ②Amelioration of drug-induced toxicities such as cachexia, pulmonary fibrosis and oxidative stress	(37–42)
Astragalus membranaceus	Astragaloside IV	miR-181a-3p/UPR-ERAD, AKT、mTOR, NF-κB, SIRT6, eNOS/NO/ONOO	Lung, prostate, breast, and liver cancer	①Enhancing the sensitivity of tumor cells to gefitinib, anlotinib, platinum and other drugs. ②Improving the drug-induced cardiotoxicity. ③Combining with various nano-delivery systems to enhance the anti-tumor effect	(48–56)
Paris polyphylla	Polyphyllin I, II, VI, VII	NRF2, PI3K/Akt, CIP2A/AKT/mTOR, MAPK	Lung, glioma, and colorectal cancer	①Enhancement of drug sensitivity and mitigation of resistance to oxitinib, cisplatin, temozolomide and adriamycin. ②Combined with nanobionic systems to enhance immunotherapy	(57–64)
Coptis chinensis Franch	Berberine	ERK1/2, P-gp/ABCB1, MRP1/ABCC1, Rap1/PI3K/Akt, EGFR, β-catenin, ER-α36, SDF-1/CXCR4,	Pancreatic, lung, ovarian, and breast cancer	①Enhance the sensitivity of tumor cells to doxorubicin, osimertinib, tamoxifen and other drugs and alleviate drug resistance. ②Improve drug-induced toxic side effects, such as pulmonary fibrosis, ototoxicity, intestinal mucositis. ③Combine with a variety of nano-delivery systems to enhance anti-tumor effect	(65–80)
Poria cocos	Pachymaran	p53	Ovarian cancer, colorectal cancer	①Enhancing the sensitivity of tumor cells to paclitaxel and 5-FU and alleviating drug resistance. ②Improving drug-induced side effects, such as weight loss and intestinal inflammation	(81–83)
Ganoderma lucidum	Ganoderma lucidum triterpenoids, Ganoderma lucidum polysaccharides	MDR-1, MRP1, TLR4/Myd88/NF-κB, Akt	Leukemia, liver cancer, and lung cancer	①Enhancing the sensitivity of tumor cells to adriamycin, cisplatin and other drugs and radiotherapy. ②Improving the drug-induced side effects, such as cognitive dysfunction and chemotherapy-related fatigue. ③Combining with nano-delivery system to enhance the anti-tumor effect	(84–92)
Semen coicis	Coix seed oil	PTEN/PI3K/AKT, miR-205/S1PR1, MuRF1/HSL/AMPK	Pancreas, colorectal, breast, and liver cancer	①Promotes apoptotic processes in a wide range of tumor cells. ②Enhanced sensitivity to drugs such as gemcitabine. ③Improvement of drug-induced cachexia. ④Combining with Nano Drug Delivery System to Enhance Anti-tumor Effects	(93–102)
Kanglaite Injection	Coix seed oil	CKLF1/NF-κB	Head and neck neoplasms, Liver, lung, and pancreatic cancer,	①Enhancing drug sensitivity and improving drug resistance to cisplatin, paclitaxel, and gefitinib. ②Improvement of radiotherapy-induced adverse effects such as mucositis, gastrointestinal reactions, nephrotoxicity, leukopenia, thrombocytopenia, myelosuppression, etc.	(103–109)
Xihuang Pill	Ox jaundice, Musk, Olibanum, Myrrh	MEKK1/SEK1/JNK1/AP-1, PI3K/Akt/mTOR, cAMP/PKA, POU4F1/STAT3/NLRP3, CD133/EGFR/Akt/mTOR	Lung, breast, liver, stomach, and glioma	①Ability to modulate multiple pathways and routes to inhibit cancer progression. ②Enhancement of the efficacy of amlotinib, temozolomide, and rapamycin and promotion of apoptotic processes	(110–120)
Compound Banmao Capsule	Cantharides, senticosus, Ginseng, Rhizoma sparganii, Curcuma phaeocaulis, Bear bile powder, and Glycyrrhiza uralensis Fisch et al.	p38/JNK, PI3K/AKT	Stomach, colorectal, osteosarcoma, liver, lung, and cervical cancer	①Enhanced sensitivity to tiglio, apatinib, gefitinib and other drugs. ②Improvement of postoperative liver function, immunity and prolongation of survival time and quality of life.	(121–132)
Kanglixin Capsule	Ferula asafoetida, Aspongopus chinensis Dallas, Terminalia chebula Retz, Radix Aucklandiae, Syringa oblata Lindl, Ophiocordyceps sinensis	–	Lung, stomach, breast, and esophagus cancers	①Enhancement of sensitization to paclitaxel, pemetrexed, cisplatin, etc. and sensitization to radiotherapy. ②Improve patients’ immune function, alleviate clinical symptoms, reduce the toxic side effects of chemotherapy drugs, and delay tumor progression	(133–141)
Xiaoaiping Injection	Marsdenia tenacissima	PXR, Bcl-2, ATF3, VEGF/MMP9/EMT	Lung, ovary, breast, and stomach cancer	①Enhanced sensitivity to paclitaxel, tigecycline, oxaliplatin, etc. ②Improvement of disease-associated malignant ascites and thrombocytopenia, delayed tumor progression	(142–154)
Elemene Injection	Curcuma phaeocaulis	–	Prostate, breast, liver, and lung cancer	①Enhancement of zoledronic acid, platinum drug sensitivity and sensitizer for radiotherapy. ②Enhancement of the body’s immunity, prolonging the survival of patients	(155–163)
Weimaining Capsule	Golden Buckwheat	–	Breast and lung cancer	①Enhanced sensitization to cisplatin, ositinib, capecitabine, etc. and sensitization to radiotherapy. ②Enhancement of immune function and improvement of inflammatory microenvironment, prolongation of progression-free survival of patients	(164–169)

In addition, we chose a few widely used anticancer CPMs for clinical observation, data presentation, and adjuvant treatment of adverse reactions to anticancer treatment and symptom relief (**Table 2**). Compared with the pure application of mainstream allopathic medicine, the anticancer treatment modality of CPM combined with mainstream allopathic medicine can significantly reduce its induced drug toxicity and related adverse reactions and improve clinical immune-related indices, tumor markers, and chemotherapy tolerance. The quality of life of patients has significantly improved. In addition to regulating tumor malignancy progression and lesion shrinkage, combined treatment places greater emphasis on the protection and physiological and psychological makeup of patients.

**Table 2:** Clinical trials of adjuvant therapy with Chinese patent medicines to reduce complications and side effects during chemotherapy.

Name	Patients (n)	Experimental group	Control group	Outcomes	*Ref.*
Kanglaite Injection (KLT)	n=1046	KLT + EGFR-TKI	EGFR-TKI	Combination significantly reduced the incidence of EGFR-TKI induced drug toxicity (nausea and vomiting)	(105)
KLT	n=554	KLT + Gefitinib	Gefitinib	Combination therapy has been shown to be effective in increasing the objective remission rate, improving physical status, and strengthening the body’s immunity	(106)
KLT	n=27	KLT + Platinum	Platinum	Combination therapy improves cellular immunity and reduces severe toxicity caused by chemotherapy	(107)
KLT	n=960	KLT + Radiotherapy and Chemotherapy	Radiotherapy and Chemotherapy	Gastrointestinal reactions, nephrotoxicity, leukopenia, thrombocytopenia, myelosuppression, and other radiotherapy-related adverse effects were reduced in patients treated with KLT	(108)
KLT	n=46	KLT + Head and neck radiotherapy	Head and neck radiotherapy	Patients treated with KLT have a low incidence of grade 3–4 radiolucent mucositis, no serious acute toxic events, and good nutritional status and quality of life	(109)
Xiaoaiping Injection (XAP)	n=86	XAP + Gefitinib	Gefitinib	Combination therapy improves blood perfusion to tumor lesions in patients with advanced lung cancer, blocks tumor vascular proliferation, and reduces tumor cell blood supply	(145)
XAP	n=173	XAP + Gefitinib	Gefitinib	Combination therapy improves patients’ coagulation function, corrects imbalances in the coagulation system, and reduces the risk of thrombosis	(146)
XAP	n=89	XAP + Epirubicin	Epirubicin	Combination therapy reduces serum tumor marker levels and improves patient immune function and quality of life	(149)
XAP	n=89	XAP + CapeOX	CapeOX	Significantly higher disease efficacy rate and disease control rate, and significant improvement in adverse effects in the combination group	(151)
XAP	n=84	XAP + SOX	SOX	Significant tumor regression and improved quality of life after combination therapy	(152)
XAP	n=140	XAP + Chemotherapy	Chemotherapy	Platelet counts in the XAP group show an upward trend and improve chemotherapy-induced thrombocytopenia	(154)
Compound Banmao Capsule (CBMC)	n=78	CBMC + TFL	TFL	Combination therapy can effectively enhance body immunity and reduce tumor markers	(122)
CBMC	n=85	CBMC + mFOLFOX6	mFOLFOX6	Combination therapy effectively improves clinical symptoms, autoimmunity and chemotherapy tolerance, and quality of life in patients with advanced colon cancer	(123)
CBMC	n=60	CBMC + S-1	S-1	Combination therapy significantly improves immune function and reduces CEA, VEGF, and CA199 levels, providing quality of life for patients	(124)
CBMC	n=80	CBMC + TACE	TACE	CBMC combined with TACE significantly improves treatment efficacy and enhances patient immune function	(127)
CBMC	n=320	CBMC + Supportive care	Supportive care	Application of CBMC prolongs survival time and increases survival benefit in patients with hepatocellular carcinoma combined with PVTT	(128)
CBMC	n=96	CBMC + Radiotherapy and Chemotherapy	Radiotherapy and Chemotherapy	Postoperative radiotherapy combined with CBMC adjuvant therapy for cervical cancer improves patients’ immunity, lowers tumor markers, and improves anti-tumor efficacy	(129)
CBMC	n=62	CBMC + Gemcitabine and Cis-platin (GP)	GP	Combination therapy increases CD4+ and CD8+ levels in the patient’s peripheral blood and improves cellular immunity in the body	(131)
CBMC	n=104	CBMC + Gefitinib	Gefitinib	The combination therapy significantly increased treatment efficacy and improved patients’ quality of life and immune function	(132)
Kanglixin Capsule (KLX)	n=60	KLX + SOX	SOX	Combined application of KLX in the treatment of intermediate and advanced gastric cancer can improve the efficiency of recent treatment, and has a positive effect in improving the immune function of patients and reducing the adverse effects in various aspects	(133)
KLX	n=80	KLX + FOLFOX4	FOLFOX4	Better results in tumor markers, body immunity, and KPS scores with the addition of KLX adjuvant therapy	(134)
KLX	n=74	KLX + DC - CIK	DC - CIK	KLX combined with DC - CIK can significantly improve the immune function of patients with advanced gastric cancer, alleviate clinical symptoms and reduce the toxic side effects of chemotherapeutic drugs	(136)
KLX	n=60/108	KLX + Pemetrexed and cisplatin (PC)/Paclitaxel and cisplatin (TP)	PC/TP	The combination of KLX can effectively prolong the survival time and improve the quality of life of patients, and has fewer adverse effects and is safe	(137, 138)
KLX	n=96	KLX + Taxol	Taxol	KLX is effective in prolonging progression-free survival, lowering tumor marker levels, and reducing the incidence of adverse effects of chemotherapy	(140)
KLX	n=60	KLX + Radiotherapy	Radiotherapy	Significantly higher overall treatment efficacy in the KLX combined radiotherapy group	(141)
Elemene Injection (EI)	n=30	EI + Radiotherapy	Radiotherapy	Combination therapy provides faster relief of clinical symptoms and improvement in leukopenia	(158)
EI	n=40	EI + Zoledronate	Zoledronate	EI has a significant potentiating effect on zoledronic acid with higher clinical efficacy rates	(161)
EI	n=543	EI + TACE	TACE	Objective remission rate and 1-year survival significantly improved in the EI + TACE group	(162)
EI	n=1410	EI + Platinum-based Chemotherap (PBC)	PBC	EI enhances the efficacy of PBC treatment and strengthens the body’s immunity	(163)
Weimaining Capsule (WMN)	n=60	WMN + Osimertinib	Osimertinib	WMN in combination with ositinib further improves disease control in patients with EGFR T790M-mutated advanced lung adenocarcinoma	(166)
WMN	n=134/188	WMN+Gemcitabine and Cis-platin (GP)	GP	WMN restores the immunosuppression produced by the GP regimen and inhibits the undesirable progression of the inflammatory microenvironment, reduces complications and decreases postoperative recurrence	(167, 168)
WMN	n=100	WMN + Capecitabine	Capecitabine	No patient experienced grade IV or higher adverse effects after combination therapy, and no patient discontinued treatment completely because of adverse effects, with excellent safety and tolerability	(169)

Chinese herb therapy has a long history and a wealth of clinical expertise. It is currently a significant component of CAM in the field of oncology, particularly in managing unpleasant medication reactions and enhancing quality of life, where it plays an indispensable role. However, there are also certain shortcomings and restrictions. CHMs have multitarget properties and can effectively counteract drug resistance in tumor therapy, but the current scientific evidence on these therapeutic means is still limited. At present, most of the studies on the antitumor mechanism of CHM are focused on *in vitro* cellular experiments, and studies on the direct intervention of CHM *in vivo* experiments and clinical observations are still in the initial stage, and many other mechanisms are not very clear. The same monomer may have an effect on multiple targets, which is not necessarily unidirectional, and the final result needs to be synthesized from a macroscopic point of view. Problems such as the difficulty of extraction, stability and drug-forming properties of CHM and its active ingredients also suggest that it has a long way to go before it becomes a clinical drug. The development of new drug delivery systems has enabled traditional natural drugs to overcome various adverse mechanisms. It can effectively improve the key issues of stability, solubility, bioavailability and pharmacokinetics in drug delivery. Still, their application is limited to a few CHMs, and the clinical promotion of CHMs and their active ingredients is still facing great challenges.

CPM is a product of the combination of modern technology and traditional drugs and has significant effects and advantages in improving patients’ clinical symptoms. The drug composition of CPM is complex, contrary to the research on CHM, which mostly focuses on *in vivo* experiments and clinical observations and lacks specific target beneficiary populations and targeted mechanisms of action. The results of mechanistic studies at the molecular level are still unclear, and some of these studies are methodologically flawed and the relevant basic research is still incomplete, limiting the advantages of multitargeting and multifunctional drugs. The advantages of CHM and CPM differ, the joint application of complementary means of TCM and mainstream allopathic medicine is beginning to be recognized, and the expanded application of novel drug delivery technologies and increasingly effective antitumor regimens with multidisciplinary combination therapy have yet to be further explored and implemented. More high-quality clinical trials are still needed to clarify the safety, efficacy, and scope of the application of CHM or CPM before they can be used for clinical treatment.

Consequently, to address the research status quo mentioned above, we must address and resolve the following issues (1): Utilizing and expanding the multitarget advantageous features of CHM and continuing in-depth exploration and in vivo validation of the antitumor mechanisms of other natural medicines and TCM monomers are highly important for improving the MDR test methodology and the development and application of TCM reversal agents and chemotherapeutic sensitizers (2); continuing to clarify the multitarget mechanism of action of CPM, improving the methodology of CPM, and making the application of CPM more precise and systematic. Moreover, we will accelerate the research and development of new antitumor drugs, devote ourselves to improving the adverse reactions and clinical symptoms caused by the antitumor process, and explore increasingly efficient CPMs (3). We will continue to explore more efficient CHM and CPM targeting protocols using advanced drug delivery technologies, carrier technologies and nanotechnology utilizing modern pharmaceuticals and accelerate the realization of clinical applications of new technologies (4). We will continue to explore more efficient CHM and CPM targeting protocols and clinical formulation carriers by utilizing advanced drug delivery technology, carrier technology and nanotechnology in modern pharmacies and accelerate the realization of clinical applications of new technologies

Chinese herb therapy is an important part of CAM and, as an important supplement to mainstream allopathic medicine, has broad application prospects, promoting therapeutic effects and improving the MDR of mainstream allopathic medicine, reducing the adverse events and mental burden of antitumor therapy, improving the quality of life of patients and prolonging survival time, and providing new interpretations and supplements to antitumor therapy at the humanistic level. It is hoped that this review can provide a new theoretical basis for its application in antitumor therapy, provide favorable research directions and ideas for the development of new antitumor drugs, and provide better, safer, and more effective therapeutic regimens and medication choices for future clinical antitumor treatments.

## Author contributions

BN: Writing – original draft, Writing – review & editing. KX: Writing – original draft. JW: Writing – review & editing. JZ: Conceptualization, Data curation, Investigation, Methodology, Writing – review & editing. LW: Investigation, Writing – original draft. XW: Data curation, Software, Writing – original draft. TL: Data curation, Writing – review & editing. NY: Funding acquisition, Writing – review & editing. JJ: Conceptualization, Data curation, Formal analysis, Funding acquisition, Investigation, Methodology, Project administration, Resources, Software, Supervision, Validation, Visualization, Writing – original draft.
